# Reduced Lamin A/C Does Not Facilitate Cancer Cell Transendothelial Migration but Compromises Lung Metastasis

**DOI:** 10.3390/cancers13102383

**Published:** 2021-05-14

**Authors:** Francesco Roncato, Ofer Regev, Sara W. Feigelson, Sandeep Kumar Yadav, Lukasz Kaczmarczyk, Nehora Levi, Diana Drago-Garcia, Samuel Ovadia, Marina Kizner, Yoseph Addadi, João C. Sabino, Yossi Ovadya, Sérgio F. de Almeida, Ester Feldmesser, Gabi Gerlitz, Ronen Alon

**Affiliations:** 1 Department of Immunology, Weizmann Institute of Science, Rehovot 76100, Israel; francesco.roncato@weizmann.ac.il (F.R.); ofer.regev@weizmann.ac.il (O.R.); sara.feigelson@weizmann.ac.il (S.W.F.); sandeepyadav.bhu@gmail.com (S.K.Y.); samuel.ovadia@weizmann.ac.il (S.O.); marina.kizner@weizmann.ac.il (M.K.); 2 Department of Molecular Biology, Ariel University, Kiryat Hamada, Ariel 40700, Israel; lukaszka@ariel.ac.il (L.K.); nehorale@ariel.ac.il (N.L.); gabige@ariel.ac.il (G.G.); 3 Department of Biological Regulation, Weizmann Institute of Science, Rehovot 76100, Israel; diana.drago@weizmann.ac.il; 4 Life Sciences Core Facilities, Weizmann Institute of Science, Rehovot 76100, Israel; yoseph.addadi@weizmann.ac.il (Y.A.); ester.feldmesser@weizmann.ac.il (E.F.); 5 Instituto de Medicina Molecular João Lobo Antunes, Faculdade de Medicina da Universidade de Lisboa, 1649-028 Lisboa, Portugal; joaosabino@medicina.ulisboa.pt (J.C.S.); sergioalmeida@medicina.ulisboa.pt (S.F.d.A.); 6 Department of Molecular Cell Biology, Weizmann Institute of Science, Rehovot 76100, Israel; yossiov@gmail.com

**Keywords:** cancer metastasis, nucleus, diapedesis, extravasation, epigenetics, chemotaxis, imaging

## Abstract

**Simple Summary:**

The nucleus is the largest and stiffest organelle of tumor cells. Cancer metastasis depends on the ability of cancer cells circulating in the blood to exit blood vessels and survive in target organs. The roles of the shell (lamina) of the nucleus in cancer cell migration and survival in distinct organs of metastasis are still unclear. A-type lamins are key lamina components that increase nuclear stiffness and reduce squeezing capacity through highly rigid barriers. We addressed how reduced expression of A-lamins (lamin A/C) affects cancer cell survival and crossing of endothelial barriers and lung capillaries and found that reduced lamin A/C expression impairs cancer growth in spheroids and restricts cancer metastasis to lungs without improving cancer cell squeezing and extravasation from lung vessels, the key platform for cancer entry into lungs.

**Abstract:**

The mechanisms by which the nuclear lamina of tumor cells influences tumor growth and migration are highly disputed. Lamin A and its variant lamin C are key lamina proteins that control nucleus stiffness and chromatin conformation. Downregulation of lamin A/C in two prototypic metastatic lines, B16F10 melanoma and E0771 breast carcinoma, facilitated cell squeezing through rigid pores, and reduced heterochromatin content. Surprisingly, both lamin A/C knockdown cells grew poorly in 3D spheroids within soft agar, and lamin A/C deficient cells derived from spheroids transcribed lower levels of the growth regulator *Yap1*. Unexpectedly, the transendothelial migration of both cancer cells in vitro and in vivo, through lung capillaries, was not elevated by lamin A/C knockdown and their metastasis in lungs was even dramatically reduced. Our results are the first indication that reduced lamin A/C content in distinct types of highly metastatic cancer cells does not elevate their transendothelial migration (TEM) capacity and diapedesis through lung vessels but can compromise lung metastasis at a post extravasation level.

## 1. Introduction

The nucleus is the largest and stiffest organelle in all cells and therefore imposes the main barrier for cell crossing of cellular and mechanically resistant extracellular barriers [1,2]. The nucleus must undergo various shape changes during cell migration through cellular and extracellular barriers [1]. A-type lamins, namely lamin A and its splice variant lamin C (herein lamin A/C), are key nuclear lamina intermediate filament proteins that control nucleus stiffness [2,3,4] and regulate chromatin conformation and accessibility [5,6]. A-type lamins also control nuclear crosstalk with all types of the cell cytoskeleton, including microtubules and actin filaments [7,8,9], and thereby regulate nuclear location and response to mechanical signals from the extracellular environment [4,10,11,12].

How nuclear squeezing is regulated in solid cancer cells migrating through variable interstitial ECM barriers and constricted vascular spaces is only partially understood [13,14,15]. The soft nuclei of most leukocytes contain low levels of lamin A/C and high levels of other lamins, primarily of the B type [4]. The low ratio of A and B lamins allows leukocytes to undergo massive and rapid deformation during fast squeezing through vascular endothelial junctions and collagenous interstitial spaces [16]. In contrast to leukocyte nuclei which express very low levels of lamin A/C, the nuclei of invasive metastatic cancer cells of mesenchymal origin contain high levels of these lamins and are therefore generally more stiff, potentially imposing higher restrictions on the ability of these cells to cross physiological barriers [17,18]. However, the cytoskeleton of endothelial cells which comprise the major barriers for nuclear squeezing during TEM is contractile [18,19]. Although collagenous barriers can undergo extensive remodeling by cell-generated forces and proteolysis [20], solid tumor cells with high lamin A/C content might less efficiently cope with these barriers than with endothelial barriers [16]. Support for this idea was recently provided by experiments with leukocytes overexpressing lamin A; a 10-fold increase in the ratio of lamin A to lamin B dramatically restricted leukocyte nuclear squeezing through rigid pores (transwell migration assays) and dense collagenous barriers but was largely permissive for leukocyte transendothelial migration [16].

The direct contributions of lamins to cancer cell migration, growth, and malignancy have been in debate [21,22]. There is no overall pattern of A-type lamin expression in cancers, and inconsistent patterns are frequently observed between cancer subtypes [23]. On one hand, lamin A/C expression is reduced in several solid cancers, and cancer progression was suggested to correlate with lower lamin A/C expression [21,22,23,24]. Decreased lamin A/C expression is also a sign of poor prognosis in several skin, breast, lung and colon cancers [24,25,26,27]. On the other hand, lamin A/C upregulation has also been shown to be associated with more aggressive cancers [23]. For instance, in pre-metastatic colon adenocarcinoma, higher lamin A levels correlate with increased motility and invasiveness and in elevated mortality in colorectal cancers [28]. Furthermore, overexpression of A-type lamins in some cancer cells causes enhanced growth, invasion, and migration by activation of the PI3K/AKT/PTEN pathway [29]. Because lamin A/C knockdown nuclei can be more easily deformed and preferentially squeeze through rigid confinements, it has been postulated that tumor cells with low expression levels of these lamins can more readily invade tissues [24,25]. Nevertheless, the direct in vivo evidence for this assumption has never been provided due to the complex roles of A-type lamins in protecting the nuclei from mechanical nuclear rupture, DNA damage, and cell growth arrest [30]. Interestingly, higher expression of the *LMNA* gene correlates with poor survival of melanoma and a subset of breast cancer patients in the TCGA dataset (Figure S1). Because poor survival is often the result of higher metastatic burden, these data prompted us to asses at the molecular level the outcome of reduced type A lamin levels on the ability of murine melanoma and breast cancer cells to give rise to cancer metastasis in syngeneic immunocompetent mice.

In the present work we have systematically tested, both in vitro and in vivo, if downregulation of lamin A/C expression with retained levels of B lamins alters specific migratory, proliferative, and metastatic properties of two bona fide murine metastatic cells, namely, B16F10 melanoma and the luminal B E0771 breast carcinoma. We specifically wished to address whether extravasation of these cancer cells from different blood vessels, and in particular from the relatively impermeable lung capillaries of syngeneic recipient mice, are favored by the softening of their nuclei introduced via downregulation of lamin A/C. In vitro, downregulated lamin A/C levels introduced by ectopic expression of lamin A/C specific shRNA dramatically facilitated the squeezing of both cells through rigid pores. Surprisingly, however, the endothelial crossing of lamin A/C knockdown cells in vitro, as well as their emigration from the pulmonary circulation were not compromised. The ability of both lamin A/C low melanoma and breast cancer cells to generate metastatic lesions in the lungs was markedly compromised. In vitro, both B16F10 melanoma and E0771 luminal B breast adenocarcinoma cells [31] with lamin A/C knockdown exhibited proliferation defects when grown in spheroids within 3D environment. Our results suggest that reduced nuclear lamin A/C content does not elevate the squeezing ability of melanoma and breast cancer cells through physiological endothelial barriers but results in impaired in vitro growth in spheroids, associated with reduced Yap1 transcription, and in slower metastatic growth of these cells in the lungs.

## 2. Materials and Methods

### 2.1. Cells

Murine melanoma (B16F10) and Lewis Lung Carcinoma (LL/2) cells were grown in DMEM supplemented with 10% FBS. Murine breast adenocarcinoma cells (E0771) were grown in DMEM supplemented with 10% FBS, 1 mM sodium pyruvate and 10 mM HEPES. Human embryonic kidney (HEK293T) and murine brain endothelial (bEnd.3) cells were cultured in DMEM medium supplemented with 10% FBS and 2 mM L-glutamine.

### 2.2. Mice

Wild-type mice (WT) on C57BL/6 background were maintained in a pathogen-free facility and all animal procedures were approved by the Animal Care and Use Committee of the Weizmann Institute of Science. Male and female 7 to 8-week-old mice were used in all experiments.

### 2.3. Imaging and Analysis of Tumor Cell Transendothelial Migration

The transmigration assay of tumor cells was performed under shear-free conditions. Murine endothelial bEnd.3 cells (8 × 10^4^) were seeded in a μ-Slide VI0.4 ibiTreat (ibidi), pre-coated with gelatin (1% in DDW) for 30 min at 37 °C. A day later, B16F10 or E0771 cells were labeled with 20 µM Hoechst 33342 for 5 min at 37 °C and resuspended in binding medium (Hank’s balanced-salt solution 1× containing 2 mg/mL BSA and 10 mM HEPES, pH 7.4, supplemented with 1 mM CaCl_2_ and 1 mM MgCl_2_) and introduced in the ibidi chamber over a confluent bEnd.3 monolayer. Images were acquired at a rate of one frame every 4–5 min for 4 h using an IX83 inverted microscope (Olympus Corporation, Tokyo, Japan) equipped with UPlanFLN 20 ×/0.50 Ph1 ∞/0.17/FN 26.5 objective (Olympus Corporation, Tokyo, Japan), 49000-ET-DAPI filter set (Chroma Technology Corp., Bellows Falls, VT, USA). ORCA-Flash4.0LT camera, model: C11440-42U (Hamamatsu Photonics K.K., Hamamatsu, Japan). Temperature was maintained at 37 °C throughout the assay. For analysis of migratory categories, tumor cells in different fields of view (10–15 cells per field) were individually tracked and categorized using cellSense software 1.16 (Olympus Corporation, Tokyo, Japan). Close monitoring of individual frames allowed the discrimination of transmigrating tumor cells from tumor cells that failed to complete TEM either because of inability to protrude through endothelial junctions (SA) or squeeze their nuclei through these junctions and underneath the endothelial monolayer (SEP). See also Video S2. Of note, we did not observe intercalation of individual tumor cells in between ECs [32]. Fiji software was used to determine nuclear circularity of transmigrating tumor cells at different frames of the time-lapse videos. This software was also used to incorporate labels, scale bars, and to manually outline tumor cell’s leading edge and nucleus into different video segments.

### 2.4. Transwell Migration Assays

Fibronectin (1.5 µg/mL in PBS) was coated for 30 min at 37 °C onto both sides (for chemotaxis assays) or only on the bottom side (for haptotaxis assays) of 8 or 3 µm hanging cell culture inserts (MCEP24H48 and MCSP24H48, Millipore, Darmstadt, Germany). After washing the filters with PBS, B16F10 or E0771 cells (4 × 10^4^) resuspended in DMEM containing 0.1% BSA were introduced into the top chamber. DMEM with 0.1% BSA was placed in the lower chamber in the presence or absence of HGF, a potent chemoattractant for B16F10 but not for E0771 cells. After 4 or 24 h at 37 °C with CO_2_, cells were fixed with paraformaldehyde (4% in PBS) for 15 min and stained with crystal violet (3% in DDW) for additional 15 min, both at RT. Cells on the upper side of the filter were scraped using a cotton swab whereas cells located on the bottom side were imaged using a SZX16 stereo microscope (Olympus Corporation, Tokyo, Japan) equipped with SDF PLAPO 1XPF objective (Olympus Corporation, Tokyo, Japan) set at 10× magnification and a DP73 camera (Olympus Corporation, Tokyo, Japan). The number of migrating cells in 5 fields of view (704 × 528 µm) per transwell insert was manually quantified using cellSense software.

### 2.5. Generation of shRNA—Expressing Cancer Cell Lines

Lentiviruses were produced by co-transfecting HEK293T cells with the shControl or shLmna vectors and three helper plasmids (Gag-Pol, Rev and VSV-G,) using Lipofectamine^®^ 2000. The virus-containing medium was harvested 48 or 72 h after transfection and subsequently precleaned by a brief centrifugation at 600× *g* and a 0.45 µm filtration. Viruses were collected and concentrated with a precipitation solution (40% PEG8000 and 2.5N NaCl) and stored at −20 °C overnight. A day later, the medium was thawed and centrifuged at 2400× *g* for 30 min at RT. The viral pseudoparticles were resuspended in 200 µL of culture medium and mixed with 2.5 × 10^4^ of tumor cells for 12 h. 36 h after viral transduction, puromycin was added to the culture medium at a concentration of 2.5 µg/mL (B16F10) or 3 µg/mL (E0771) and tumor cells were selected and expanded, replacing growth media every 48 h. Mean protein knockdown levels were assessed by Western blotting. Detailed information about the western blot can be found at Figure S14.

### 2.6. RNA Isolation and Real—Time qRT-PCR

RNA was isolated using Total RNA Mini Plus (A&A Biotechnology, Gdańsk, Poland). Complementary DNA (cDNA) was synthetized using qScript^TM^ cDNA Synthesis Kit (Quantabio, Beverly, MA, USA). SYBR Green real time—PCR was carried out according to the manufacturer’s instructions (Applied Biosystems, Waltham, MA, USA). qPCR signals (cT) in each experimental group were normalized to GAPDH levels.

### 2.7. Imaging of Tumor Cell Nuclear Dynamics

B16F10 or E0771 cells (2 × 10^4^) were trypsinized, labeled in suspension with 20 µM Hoechst 33342, resuspended in binding medium (composition described above), and introduced in a μ-Slide VI0.4 ibiTreat (ibidi) over a bEnd.3-deposited basement membrane extracellular matrix. Images were acquired at a rate of one frame every 4–5 min for 2 h using an IX83 Inverted Microscope (described above). Temperature was kept at 37 °C throughout the assay. Background was subtracted for the fluorescent channel using cellSense 1.16 (Olympus Corporation, Tokyo, Japan) software. FiJi (SciJava) software was used for title and time code labeling and determination of nuclear circularity of individual cells.

### 2.8. Light Sheet Fluorescent Microscopy of Tumor Cells and Lung Vasculature

B16F10 (2 × 10^4^) or E0771 (10^4^) cells labeled with CMTMR dye (Thermo Fisher Scientific), 10 µM for 30 min according to the manufacturer’s instructions, were injected in the retro-orbital sinus of recipient mice. Euthanasia by administration of sodium pentobarbital (200 mg/Kg) was practiced 3 h later. Blood capillaries were labeled 15 min before the animal sacrifice by intravenous injection of 6 µg of an Alexa 647-conjugated anti-CD31 mAb. Immediately after the sacrifice, mice were transcardially perfused with PBS and the lungs inflated via the trachea with low gelling agarose (Sigma-Aldrich, Darmstadt, Germany), subsequently fixed with paraformaldehyde (4% in PBS) for 2 h, dehydrated and cleared using ethyl cinnamate as described in [33]. Cleared intact lung lobes, were imaged using an Ultramicroscope II (LaVision BioTec, Bielefeld, Germany) operated by the ImspectorPro software (LaVision BioTec, Bielefeld, Germany). For excitation light sheet was generated by a Superk Super-continuum white light laser (emission 460 nm–800 nm, 1 mW/nm–3 (NKT Photonics, Birkerød, Denmark), followed by specific excitation filters per channel. For detection optics microscope was equipped with a single lens configuration-4× objective-LVBT 4X UM2-BG, with an adjustable refractive index collar set to the RI of 1.56. Images were acquired by an Andor Neo sCMOS camera (2560 × 2160, pixel size 6.5 µm × 6.5 µm, Oxford Instruments, Abingdon, Oxfordshire). Z stacks were acquired in 3 μm steps. Channel configuration for GFP and EGFP excitation 470\40 emission 525\50, for CMTMR, excitation 560\40 emission 630\75, and for CD31-AF647 excitation 640\30 emission 690\50.

### 2.9. Image Reconstruction and Analysis

Three-dimensional rendering of LSFM was performed via Imaris software (Oxford Instruments, Abingdon, Oxfordshire, UK). Surfaces of CMTMR-labeled tumor cells were created using volume (comprised between 1000 and 25,000 µm^3^) and intensity (max of red fluorescent channel) as defining features to unequivocally separate them from background signals. Each cell was individually segmented and its distance was measured with respect to the CD31-labeled blood vessels. Cell positions of intravascular, extravascular or protruding were determined by this spatial analysis. See also Videos S6–S8.

### 2.10. Determination of Tumor Cell Accumulation in Lungs

B16F10 (2 × 10^4^) or E0771 (10^4^) cells labeled with 10 µM CMTMR for 30 min according to the manufacturer’s instructions, were resuspended in PBS and injected into the retro-orbital sinus of recipient mice. When long-term survival and lung metastasis were determined i.e., >3 days post injection, cancer cells were introduced through tail vein injection to avoid the formation of eye tumors at the site of injection. Euthanasia by administration of sodium pentobarbital (200 mg/Kg) was practiced 3 h or 3–7 days later. Immediately thereafter, mice were transcardially perfused with PBS and the lungs were extracted, minced and incubated in RPMI-1640 containing collagenase type 4 (1.5 mg/mL) and DNase I (20 µg/mL) at 37 °C for 45 min. Lung cell suspensions were pushed through a 100 µm cell strainer and centrifuged at 0.2× *g* or 5 min at 4 °C. RBCs were subsequently lysed with an RBC lysis buffer (Sigma-Aldrich, Darmstadt, Germany). The cells were resuspended in ice-cold FACS buffer (PBS with 1% BSA, 0.1% sodium azide and 5 mM EDTA), filtered through a 70 µm strainer. Additionally, the cell suspension was resuspended in Annexin V Binding buffer (BioLegend, San Diego, CA, USA), labeled with APC-conjugated Annexin V antibody (BioLegend, San Diego, CA, USA) for 15 min at RT and analyzed using a CytoFLEX flow cytometer (Beckman Coulter, Brea, CA, USA).

### 2.11. Cell Growth on 2D Culture Plates

B16F10 or E0771 cells were seeded at a low density of 5 × 10^3^/well in a 6-well plate. Puromycin-containing growth medium was replaced every 24 h throughout the duration of the assay (72 h). To determine the number of viable cells proliferating on the plates, cells were trypsinized and counted by flow cytometer every 24 h.

### 2.12. Cell Growth in 3D Soft Agar

B16F10 or E0771 cells were suspended at a density of 4 × 10^3^/mL of 0.3% low gelling agarose in DMEM +10% FBS and equilibrated at 4 °C for 15 min in 12-well plate as described [34]. The cell agar suspension was warmed to 37 °C and cultured in a humidified incubator containing 5% CO_2_ at either normoxic (21% O_2_) or hypoxic (1% O_2_) conditions. Spheroid formation was monitored on the first, third and 6th day using a IX83 inverted microscope (Olympus) equipped with UPlanFLN 4 ×/0.13 Ph1 ∞/-/FN 26.5 objective (Olympus). In parallel, cancer cell colonies grown directly on the bottom of each well were also imaged. To isolate individual spheroids grown in 3D conditions, the layer of agarose containing the spheroids was mechanically removed and solubilized in PBS prewarmed at 45 °C. Spheroids were spun down at 0.8× *g* for 5 min at RT and either trypsinized with trypsin B to recover single cells stained for annexin V and propidium iodide (BioLegend), or lysed to obtain total RNA. Alternatively, spheroids were settled on a glass-bottom dish previously coated with 0.01% poly-L-lysine for 30 min at 37 °C, fixed with 4% PFA in PBS for 3 h at RT, permeabilized with 1% Triton X-100 in PBS for 3 h at RT, blocked with 10% horse serum for 30 min at 37 °C and stained overnight at 4 °C with the primary antibody, followed by incubation with the secondary antibody and Hoechst 33342 for 1h at RT.

### 2.13. Experimental Lung Metastases

B16F10 (4 × 10^4^) or E0771 (10^4^ or 2 × 10^4^) either shControl or shLmna cells were suspended in 200 µL PBS +0.25 mM EDTA and injected into the tail vein of recipient mice. Animals were euthanized by administration of sodium pentobarbital (200 mg/Kg) 14 or 28 days later. Immediately after the sacrifice, mice were transcardially perfused with PBS and the lungs were extracted and visually analyzed for the presence of surface metastatic foci, subsequently stored in 4% PFA for 24 h and 1% PFA at 4 °C for long term storage. Paraffin embedding and H&E staining of 5 µm-thick sections were performed by the histology unit of the Weizmann Institute of Science. Sections were digitalized using a Pannoramic SCAN II (3DHISTECH, Budapest, Hungary) and analyzed using CaseViewer software (3DHISTECH, Budapest, Hungary).

### 2.14. Patient Survival Analysis

Kaplan-Meier curves representing patient disease specific survival (DSS), were generated using survminer R package. Maximally selected rank statistics was used to determine the optimal cutpoint for patient survival based on *LMNA* mRNA expression levels (high and low). TCGA data for metastatic skin cutaneous melanoma and luminal B invasive breast carcinoma survival and expression were retrieved using cgdsr (v.1.3.1) R package.

### 2.15. Statistical Analysis

Data in graphs are represented as mean or mean ± standard error of the mean (SEM). Student’s two-tailed unpaired *t* test or Mann-Whitney two-tailed *U* test were used to determine the significance of the difference between means of two groups. One or two-way ANOVA tests were used to compare means among three or more independent groups/categories. Significance was set to *p* < 0.05. Statistical details of experiments can be found in the figure legends.

### 2.16. Additional Supplementary Methods

Additional methods sections are included in Supplementary Material and Methods [35,36,37,38,39,40,41,42,43,44,45].

## 3. Results

### 3.1. Lamin A Downregulation in B16F10 Melanoma Increases Nucleus Deformability and Squeezing through Rigid Pores but Does Not Affect Transendothelial Migration

**Figure 1:**
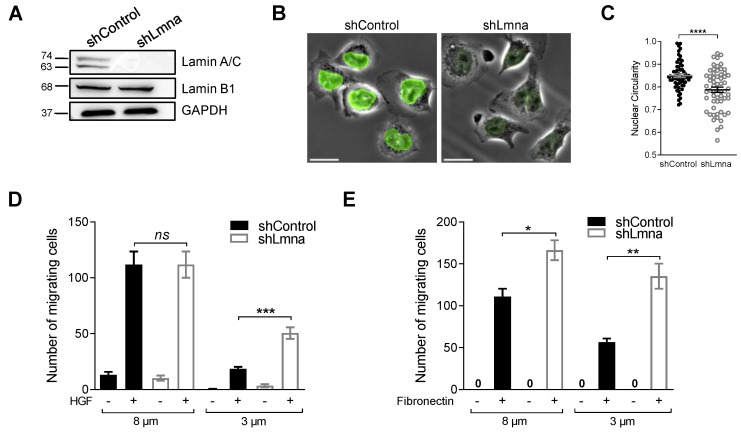
Downregulation of lamin A/C increases melanoma squeezing through small rigid pores but does not facilitate tumor transendothelial migration in vitro. (**A**) Expression levels of lamin A/C and lamin B1 in B16F10 cells transduced with control or lamin A/C shRNA. Glyceraldehyde-3-phosphate dehydrogenase (GAPDH) was used as loading control. (**B**) Representative immunostaining of lamin A/C (green), superimposed on phase contrast images of B16F10 shControl and shLmna cells. Scale bar, 20 µm. (**C**) Nuclear circularity of B16F10 shControl or shLmna cells spread on a bEnd.3-derived basement membrane (*n* = 50). See also Video S4. Data are represented as mean ± SEM. **** *p* < 0.0001, Student’s two-tailed unpaired *t* test (**A**–**C**). (**D**) Chemotactic migration of B16F10 shControl or shLmna cells through 8 or 3 µm pores transwell filters in presence (+) or absence (−) of HGF (50 ng/mL) for 4 h. Data are represented as mean ± SEM of three independent experiments. *** *p* (0.0002); *ns*: nonsignificant. (**E**) Haptotactic migration of B16F10 shControl or shLmna cells through 8 or 3 µm pores transwell filters in the presence or absence of fibronectin for 4 h. Data are represented as mean ± SEM of three independent experiments. * *p* (0.0107); ** *p* (0.0024). Student’s two-tailed unpaired *t* test (**C**,**D**).

We first downregulated lamin A and lamin C expression in the nuclei of B16F10 melanoma cells by stably expressing in these cells a lamin A/C shRNA construct targeting exon 8 of the *Lmna* gene (Figure 1A,B). Because the nuclear lamina stiffness is sensitive to the ratio between type A and type B lamins in both mesenchymal and hematopoietic cells [4], we expected that lamin A/C downregulation would also increase nuclear deformability and squeezing capacities in our melanoma cell model. Downregulating lamin A and lamin C expression in the B16F10 melanoma line by 90%, but leaving lamin B1 levels intact altered the ratio of type A to type B lamins by approximately 10-fold (Figure 1A). As expected, lamin A/C downregulation resulted in decreased nuclear circularity in a nonconfined space (Figure 1C) as well as a dramatic enhancement of B16F10 squeezing through small rigid pores, but much less so through large pores (Figure 1D,E). A similar gain of squeezing of B16F10 cells was observed by using another lamin A/C shRNA construct with weaker knockdown potency (Figure S2A, shLmna-2), and in cells subjected to transient siRNA-mediated downregulation (Figure S2B).

**Figure 2:**
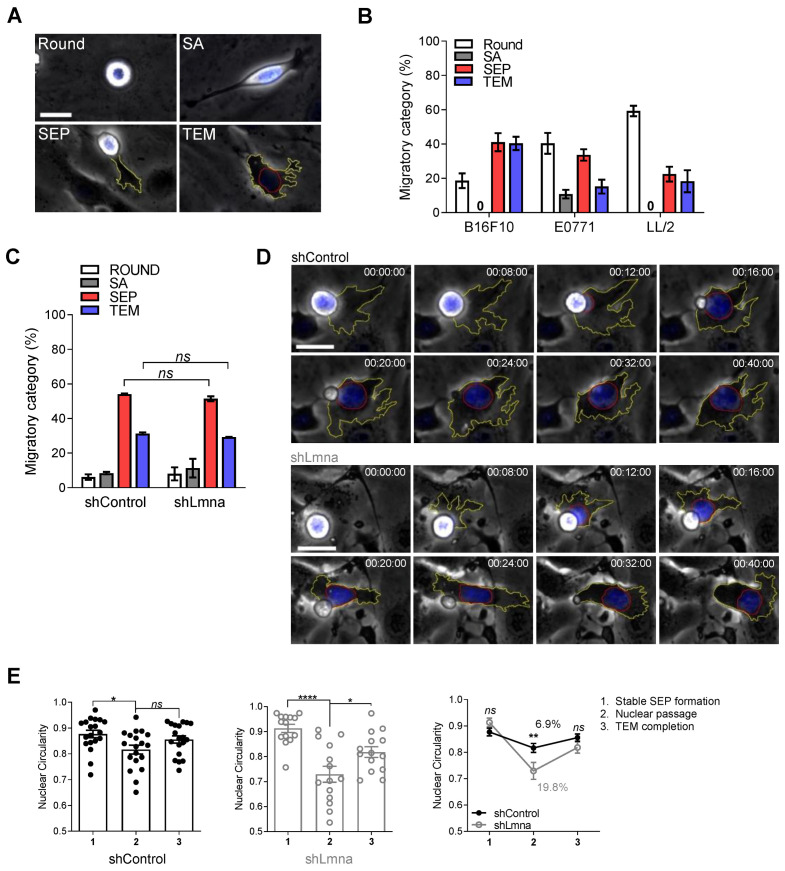
Downregulation of lamin A/C does not facilitate melanoma TEM but increases tumor nucleus deformability. (**A**) Representative images of distinct tumor cell categories (referred to as migratory categories) taken from time-lapse video microscopy segments of individual B16 cells: round, spread above (SA), forming subendothelial pseudopodia (SEP), and completing transendothelial migration (TEM). The contours of the tumor cell’s leading edge and nucleus are outlined in yellow and red respectively. Scale bar, 20 µm. See also Video S2. (**B**) Migratory categories of B16F10 melanoma, E0771 breast carcinoma and LL/2 Lewis Lung Carcinoma cells interacting with bEnd.3 monolayers (*n* = 60). Data are represented as mean ± SEM of two independent experiments. (**C**) Migratory categories of B16F10 shControl or shLmna cells interacting with unstimulated bEnd.3 cells (*n* = 60). Data are represented as mean ± SEM of three independent experiments. (**D**) Serial images of representative B16F10 control and lamin A/C knockdown cells, labeled with Hoechst 33342, during TEM. Scale bar, 20 µm. Time intervals are depicted in each image. The contours of the tumor cell’s leading edge and nucleus are outlined in yellow and red respectively. See also Video S3. (**E**) Changes of nuclear circularity during the distinct phases (1–3) of transendothelial migration of B16F10 shControl or shLmna cells (*n* = 19, shControl; *n*= 14, shLmna). Data are represented as mean ± SEM. * *p* (shControl) = 0.0190; * *p* (shLmna) = 0.0338; **** *p* < 0.0001. The right inset depicts the mean nuclear circularity ± SEM for each experimental group. The percent changes in mean circularity values are shown. ** *p* (0.0057); *ns*: nonsignificant. Two-way (**A**,**C** inset) or one-way (**C**) ANOVA with Bonferroni’s (**A**,**C** inset) or Tukey’s (**C**) post hoc tests.

To assess if and how these changes in the composition and deformability of the tumor nucleus promote tumor cell squeezing through physiological endothelial barriers, we established a new in vitro video microscopy-based assay in which nuclear squeezing of tumor cell crossing confluent monolayers of bEnd.3 murine endothelial cells can be compared. Nucleus location, deformation and squeezing in individual transmigrating tumor cells could be readily tracked in real time by fluorescence and phase contrast microscopy of tumor cells whose nuclei were prelabeled with the nuclear dye Hoechst (Videos S1 and S2). This assay allowed us to follow how individual tumor cells complete an entire sequence of transendothelial migration (TEM) steps immediately after settling on confluent endothelial monolayers, including protrusion, generation of large subendothelial pseudopodia (lamellipodia), squeezing their nuclei, tail detachment, and locomotion underneath the endothelial monolayer (Figure 2A; Video S2). Out of three murine cancer cell lines compared in this new assay, namely B16F10, E0771, and LL/2, B16F10 melanoma exhibited the highest extent of TEM (Figure 2B). Notably, the TEM of B16F10 as well as of other cancer cells compared in this system was over 30-fold slower than that of leukocytes [16,19] due to a very slow formation of the subendothelial leading edge (*t* = 26 ± 22 min for B16F10 in contrast to 30 ± 15 s for T cells) and the inability of the tumor nucleus to translocate into this leading edge as during leukocyte TEM [16,19].

Surprisingly, and in contrast to the squeezing results across rigid pores (Figure 1D,E), the extent of B16F10 TEM was not increased by lamin A/C knockdown (Figure 2C,D; Video S3). However, the extent of nuclear deformation for lamin A/C downregulated nuclei was significantly greater than that of the control tumor cells (Figure 2E). The nuclei of lamin A/C knockdown B16F10 cells also deformed more readily when these cells spread on a nonconfined 2D substrate coated with the basement membrane deposited by the endothelial monolayer (Figure 1C), whereas the motility of lamin A/C knockdown cells was unaltered (Video S4). These results collectively suggest that the reduced nuclear stiffness and the increased nuclear deformability of lamin A/C knockdown B16F10 cells do not provide a migratory advantage for the transmigration of these cells through endothelial barriers. These observations also suggest that differences in tumor cell squeezing through infinitely rigid barriers do not correlate with squeezing through physiological cellular barriers. This finding could be due to the high mechanical flexibility of the endothelial cytoskeletal barriers maintained by their rapid actin turnover and dynamic contractility [19].

### 3.2. Lamin A Downregulation Does Not Increase B16F10 Extravasation across Lung Vessels In Vivo and Does Not Accelerate Melanoma Apoptosis in the Lung Parenchyma

**Figure 3:**
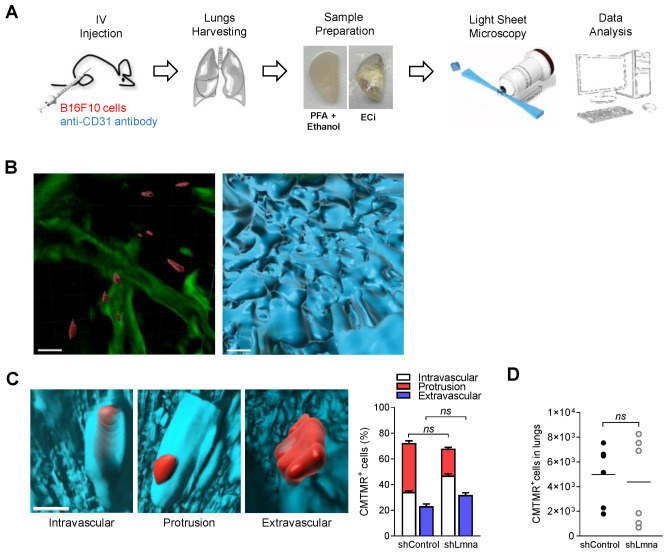
In vivo melanoma crossing of lung capillaries is not enhanced by lamin A/C downregulation. (**A**) Schematic representation of the LSFM analysis. (**B**) Visualization of 3D bronchial structures (green, autofluorescence) together with B16F10 cells (red, CMTMR) and alveolar capillaries (cyan, CD31). Scale bars, 100 μm. See also Video S5. (**C**) Representative 3D images of intravascular, extravascular, and protrusive tumor cells across the CD31-labeled lung vasculature together with the percentage of B16F10 shControl and shLmna cells present in a volume of 5 × 10^9^ µm^3^ of the left lung lobe 3 h after retro-orbital injection. Cells were counted using Imaris software (*n* = 40 cells). A representative of two experiments. Scale bar, 100 µm. See also Videos S6–S8. (**D**) The number of CMTMR-labeled B16F10 shControl or shLmna cells present in the entire lungs of recipient mice, 3 h after retro-orbital injection, quantified by flow cytometry (*n* = 6 mice). *ns*: nonsignificant. Two-way ANOVA with Bonferroni’s post hoc test (**C**), Student’s two-tailed unpaired *t* test (**D**).

Our in vitro results thus indicated that lamin A/C downregulation in B16F10 cells enhance their squeezing through rigid confinement without affecting ability to transmigrate across endothelial barriers. To assess the distinct migratory outcomes of lamin A/C downregulation in B16F10 melanoma cells in vivo, we introduced an experimental lung metastasis model based on intravenous (IV) injection of minute numbers of fluorescently labeled cells (Figure S3), in order to minimize nonphysiological inflammatory responses associated with a bolus of cancer cells that simultaneously enter the lung vasculature. Tumor metastasis in the lung spreads nearly exclusively through the pulmonary capillaries [46], an extensive network of relatively impermeable capillaries considered to be poorly permeable compared with vessels targeted by metastatic cells at other organs like the bone marrow and liver [47]. IV injections of tumor cells are extensively used for studying hematogenous dissemination and expansion in the lung [48]. Because the tumor cells are introduced in a single event, their arrival at the lung vasculature is synchronized which allows accurate temporal dissection of the earliest extravasation steps taken by individual circulating metastatic cells entering the lung vasculature several hours post injection. To determine the effects of lamin A/C downregulation on the earliest tumor cell extravasation across the lung capillaries, we compared the cancer cell partition inside and outside lung vessels with newly developed 3D imaging of the injected fluorescently labeled cancer cells in relation to CD31 stained lung vessels (Figure 3A,B; Video S5). Surprisingly, lamin A/C downregulated B16F10 cells extravasated at similar efficiencies to control B16F10 cells (Figure 3C; Videos S6–S8). Lamin A/C downregulation also did not affect the total number of B16F10 cells accumulated inside the recipient lung at these early time points (Figure 3D). These results suggest that, unlike its dramatic effects on tumor squeezing in vitro, but consistent with our TEM results in vitro (Figure 2A), lamin A/C suppression does not facilitate melanoma extravasation across lung vessels in vivo. Although, in vitro, the resistance of some lamin A/C deficient cancer cells to apoptosis induced by pulses of shear stress was reported to be lower compared to control cells [49], in our experimental setting lamin A/C B16F10 cells accumulating in the lung vasculature shortly after injection did not experience elevated cell death, as indicated by their normal recovery from total lung cell suspensions (Figure 3D).

### 3.3. Lamin A/C Downregulation Slightly Reduces the Content of H3K9Me3 Heterochromatin but Does Not Elevate Transcription

**Figure 4:**
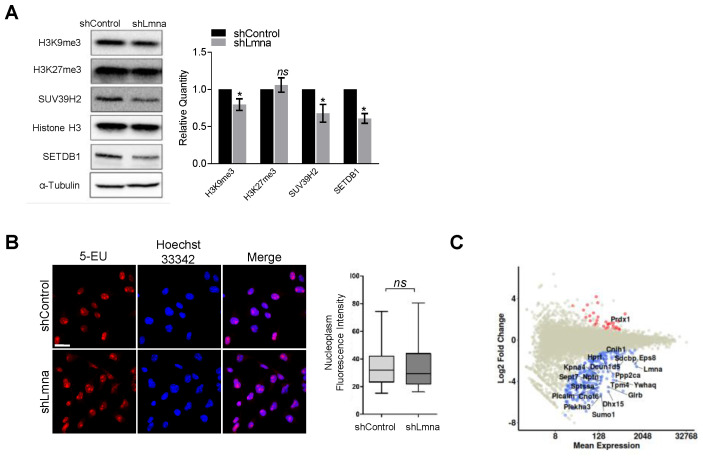
Lamin A/C downregulation reduces heterochromatin content but represses gene transcription. (**A**) Equal amounts of proteins from B16F10 shControl or shLmna cells, separated by SDS-PAGE and analyzed for the indicated proteins by Western blot analysis. The bar graph represents the mean levels of H3K9me3, H3K27me3 and SUV39H2 normalized to Histone H3 and of SETDB1 normalized to α-Tubulin ±SEM of at least four independent experiments. * *p* < 0.05; *ns*: nonsignificant. (**B**) Fluorescence microscopy imaging of 5-ethynyl uridine (EU) incorporation (red) and Hoechst 33342 (blue) in B16F10shControl and shLmna cells (*n* = 90). Data are represented as mean ± SEM of three independent experiments. Scale bar, 25 µm. (**C**) Log2FoldChange versus mean expression levels of differentially downregulated (blue), upregulated (red) and nondifferentially expressed genes (grey) are shown. The names of the top 20 most significantly expressed genes are indicated in the plot. Student’s two-tailed unpaired *t* test (**B**), Mann–Whitney two-tailed *U* test (**C**).

*Lmna* knockout was shown to reduce the major marker of constitutive heterochromatin H3K9me3 and the methyltransferases that generate it in primary cells [50]. As predicted, lamin A/C downregulation led to lower levels of H3K9Me3 and significantly reduced levels of SUV39H2 and SETDB1, two main histone methyltransferases that maintain this suppressive epigenetic marker (Figure 4A). In contrast, facultative heterochromatin levels probed by the H3K27me3 marker remained unchanged. This slight global H3K9me3 reduction was not associated with repression of transcription as determined by a transcription run-on experiment (Figure 4B). Transcription of specific genes may be altered by reduced H3K9me3 levels and lamin A/C deficiency was reported to increase chromatin dynamics [6,51]. To evaluate these possibilities, we performed RNA-Seq analysis on control and lamin A/C shRNA transduced B16F10 cells. We found that the transcript levels of LINC complex components such as nesprins 1–4, emerin, BAF as well as of other lamin A/C interactors involved in heterochromatin content and stability were not altered in lamin A/C knockdown B16F10 cells (Table S1). However, several hundred genes, including nuclear matrix genes, nuclear body genes, as well as lamin B receptor, were transcribed at lower levels in lamin A/C knockdown B16F10 cells (Figure 4C and Figure S4).

### 3.4. Lamin A/C Downregulation Does Not Alter Cell Proliferation In Vitro on 2D Surfaces but Impairs 3D Cell Growth in Spheroids

**Figure 5:**
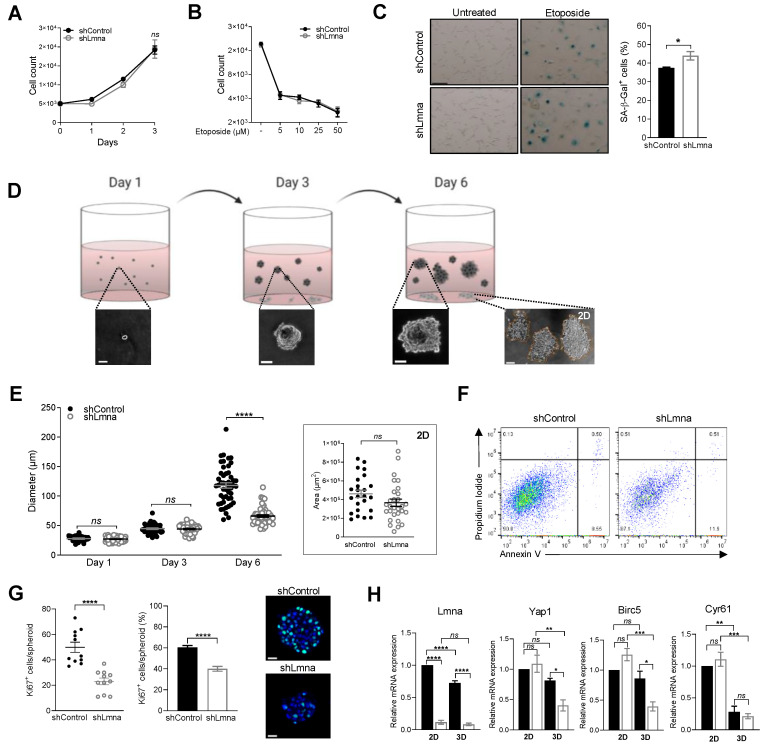
Melanoma proliferation in spheroids is reduced by lamin A/C downregulation. (**A**) In vitro cell growth of B16F10 shControl or shLmna cells. Data are represented as mean ± SEM of three independent experiments. (**B**) Growth arrest of B16F10 shControl or shLmna cells treated for 72 h with different concentrations of etoposide. Data are represented as mean ± SEM of two independent experiments. (**C**) Senescence associated β-galactosidase staining of B16F10 shControl or shLmna cells treated with 5 µM etoposide for 72 h, washed and cultured in regular growth medium for additional 120 h (*n* = 60). * *p* (0.0470). Scale bar, 20 µm. Data are represented as mean ± SEM of two independent experiments. (**D**) Schematic representation of the soft agar colony formation assay. Spheroids (dark grey) grow inside agarose while few cells migrate and start dividing at the bottom of the culture dish. Created with BioRender.com. Representative images of the spheroids derived from B16F10 shControl cells grown in 3D agar (supplemented with 10% FBS) imaged on days 1, 3 and 6. Scale bar, 20 µm. A representative image of three cell colonies proliferating on the bottom of the dish is depicted on the right. Scale bar, 200 µm. (**E**) Spheroid diameter, measured on days 1, 3 and 6 after seeding (*n* = 50). Data are represented as mean ± SEM of two independent experiments. Inset depicts the mean area ± SEM of individual 2D cell colonies proliferating on the bottom of the dish, measured on day 6 post seeding. The numbers of detectable spheroids for the two experimental groups compared were not significantly different (not shown). (**F**) Flow cytometry plots showing annexin V (X-axis) and propidium iodide (Y-axis) staining of B16F10 shControl or shLmna cells derived from 3D colonies grown in soft agar on day 6. The left lower quadrant indicates viable cells. (**G**) The left and middle panels depict, respectively, the numbers of Ki67^+^ B16F10 shControl or shLmna cells in individual spheroids isolated on day 6 and their fractions within each spheroid (*n* = 11). Representative immunostaining of spheroids for Ki67 (green), superimposed on Hoechst 33342 labeled nuclear staining (blue) is shown in the right panels. Scale bar, 20 µm. (**H**) Relative *Lmna*, *Yap1*, *Birc5* and *Cyr61* transcription levels assessed by qRT-PCR for control and lamin A/C knockdown B16 cells grown on 2D plates or within 3D spheroids isolated on day 6 post seeding. Values are relative to shControl grown in 2D. Data are represented as mean ± SEM of three independent experiments. * *p* < 0.05; ** *p* < 0.005; *** *p* < 0.0005; **** *p* < 0.0001; *ns*: nonsignificant. Student’s two-tailed unpaired *t* test (**A**–**C**,**E**,**G**) or one-way ANOVA with Tukey’s post hoc test (**H**).

Our transcriptional analysis did not detect any elevation in genes involved in melanoma growth (Table S1). Indeed, melanoma cells knockdown in lamin A/C expression exhibited similar growth rates when seeded at low densities on tissue culture plates (Figure 5A). Furthermore, deficiency in lamin A/C content also did not increase the susceptibility of the nuclei of these melanoma cells to DNA damage: lamin A/C knockdown B16F10 cells remained as resistant as control melanoma cells to apoptosis and growth arrest induced by the DNA damage and cell cycle arrest inducer etoposide [52] (Figure 5B). An additional incubation period without etoposide (Figure S5) drove the growth-arrested B161F0 cells into senescence with a slightly higher senescence acquisition exhibited by the lamin A/C knockdown melanoma cells (Figure 5C). Nevertheless, the quantity of R-loops, transient RNA–DNA hybrids which correlate with DNA damage [53], were comparable for control and lamin A/C knockdown cells (Figure S6). These results collectively suggest that lamin A/C knockdown B16F10 expand at comparable rates to control cells in conventional 2D culture conditions and do not exhibit higher susceptibility to spontaneous or biochemically induced DNA damages.

The soft agar colony formation assay has been a hallmark of cancer survival and proliferation analysis because it measures the ability of cells to proliferate in semi-solid matrices [34]. This readout also allows a direct comparison of cancer cell growth in spheroids vs. in 2D colonies expanded in the same soft agar, while remaining in direct contact with the culture dish surface (Figure 5D). Interestingly, whereas in the first three days both lamin A/C knockdown and control B16F10 cells gave rise to similar sized spheroids (Figure 5E), the ability of lamin A/C high B16F10 spheroids to further expand during the next three days significantly surpassed that of lamin A/C knockdown cells (Figure 5E). The number of colonies did not change, however. In contrast, the growth rate of both B16F10 cell groups settled on the culture plate in the presence of identical soft agar remained similar at all time points (Figure 5E, inset). These results were reproduced with another lamin A/C knockdown B16F10 line expressing a distinct lamin A/C shRNA (Figure S7A, shLmna-2). Furthermore, even in the presence of nutrient rich agar i.e., a five-fold higher serum concentration that accelerated the growth of both lamin A/C high and lamin knockdown B16F10 cells, the mean size of lamin A/C high B16F10 cells spheroids was significantly higher than that of lamin A/C low B16F10 cells (Figure S8A). The growth deficiency of the lamin knockdown cells in 3D spheroids was conserved both in standard and oxygen-deprived conditions (Figure S8B). Interestingly, only a negligible fraction of cells isolated from both control and lamin A/C low B16 spheroids (9 vs. 12 %) underwent apoptosis as depicted by annexin V and propidium iodide staining (Figure 5F), ruling out the possibility that cells growing at the core of these spheroids have poor accessibility to nutrients. The number of proliferating cells inside individual spheroids and their relative fraction were significantly reduced in the case of lamin A/C deficient B16F10 cells (Figure 5G). In agreement with their reduced growth inside spheroids, lamin A/C knockdown B16F10 cells transcribed significantly reduced levels of the transcriptional regulator *Yap1* (Figure 5H) compared to the control B16F10 cells. Notably, the reduction in this Hippo pathway factor involved in organ size control [54] was observed only in 3D spheroids of B16F10 cells and not in cells grown in the 2D cultures (Figure 5H and Figure S7B). Lamin A/C deficient B16F10 cells in spheroids also transcribed significantly lower levels of *Birc5* (survivin) a prosurvival factor controlled by *Yap1* [55]. In contrast, the same cells transcribed normal *Birc5* levels when grown on 2D surfaces (Figure 5H). Because this factor is abundantly expressed by B16F10 cells (Table S1), this result was consistent with the reduced *Yap1* transcripts of these cells within spheroids. Comparing the levels of *Cyr61* a second *Yap1* regulated proliferation factor [56] between control and lamin A/C deficient B16F10 cells, we found that the transcription of this factor was significantly reduced in both groups of cells growing inside spheroids as compared to their counterparts growing in normal 2D culture conditions (Figure 5H and Figure S7B). Collectively, our results suggest that, whereas initially, lamin A/C knockdown B16F10 proliferate at identical rates to those of control cells under both 2D and 3D conditions, the ability of lamin A/C knockdown cells to expand within 3D spheroids is significantly impaired, due to slower proliferation of these cells inside large spheroids embedded in 3D environments, in correlation with reduced *Yap1* and *Birc5* transcription.

### 3.5. Lamin A/C Deficiency Results in Reduced B16F10 Lung Metastasis

**Figure 6:**
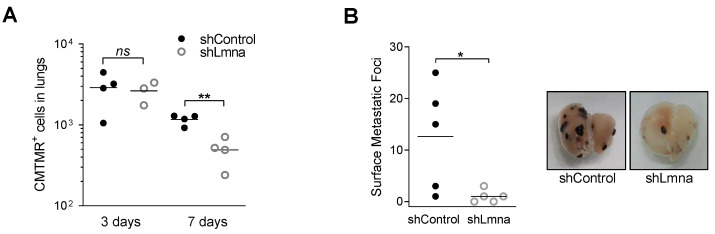
Lamin A/C downregulation does not affect early melanoma accumulation but abrogates metastatic growth in the lungs. (**A**) Number of CMTMR-labeled B16F10shControl and shLmna cells present in the lungs of recipient mice on days 3 and 7 after tail vein injection (*n* = 4; *n* = 3, shLmna on day 3). ** *p* (0.0019); *ns*: nonsignificant. (**B**) 40,000 B16F10 shControl and shLmna cells were injected into the tail vein of recipient C57BL/6 mice. After 14 days, animals were euthanized, lungs harvested and surface metastatic foci were macroscopically counted (*n* = 5). * *p* (0.0374). Representative lung images from each group are displayed. Student’s two-tailed unpaired *t* test (**A**,**B**).

In order to validate our in vitro observations, and because the diapedesis capacities of lamin A/C knockdown cells were similar to those of control cells, we next followed the survival of individual cancer cells reaching the lung parenchyma at a post extravasation level. Although lamin A/C knockdown B16F10 accumulated at comparable levels to control B16F10 inside recipient lungs within the first three days after injection, the number of lamin A/C knockdown B16F10 recovered from recipient lungs seven days after injection was significantly reduced relative to control B16F10 cells (Figure 6A). The similar recovery of lamin A/C knockdown B16F10 three days post injection into recipient mice indicated that the lamin A/C deficient cells that reached the lungs did not undergo a higher rate of apoptosis due to shear stress associated damages. Furthermore, the rate of apoptosis on day 7 post injection was low and similar for both cellular groups (Figure S9). The ability of lamin A/C B16F10 cells to generate metastatic lesions 14 days after injection into recipient mice was dramatically reduced by lamin A/C downregulation (Figure 6B). These results collectively suggest that lamin A/C knockdown B16F10 cells poorly survive in the lungs, in spite of their intact extravasation potential when circulating and entering the blood vasculature.

### 3.6. Reduced Lamin A/C Levels in E0771 Breast Cancer Cells Recapitulate the In Vitro and In Vivo Migratory Properties and Metastasis Deficiency of Lamin A/C Knockdown B16F10 Cells

**Figure 7:**
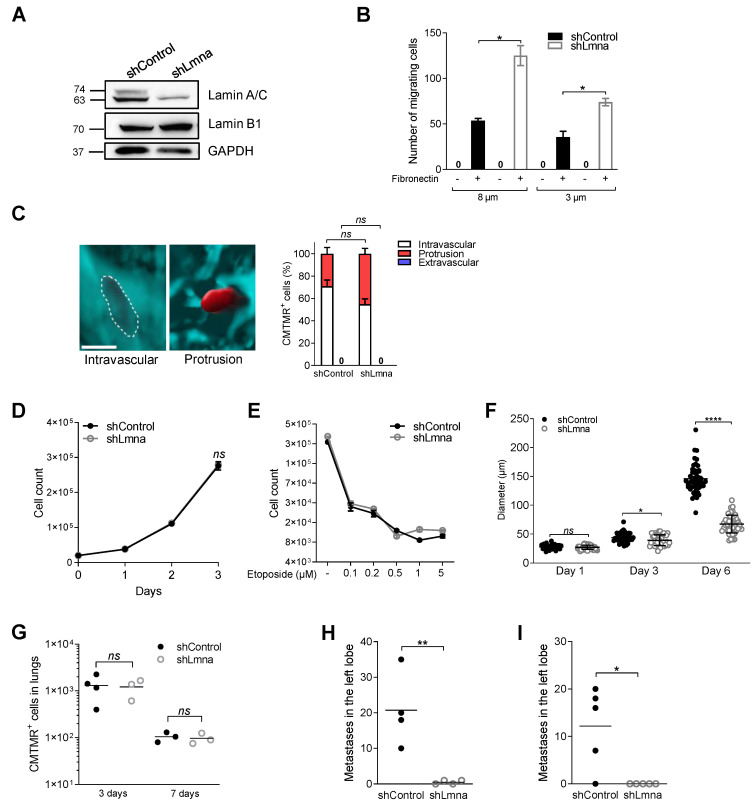
Lamin A/C downregulation in E0771 increases their squeezing capacity in vitro but reduces growth in 3D spheroids and metastatic potential in the lungs. (**A**) Expression levels of lamin A/C and lamin B1 in E0771 cells transduced with control or lamin A/C shRNA. Glyceraldehyde-3-phosphate dehydrogenase (GAPDH) was used as loading control. (**B**) Haptotactic migration of E0771 shControl and shLmna cells through 8 or 3 µm pores transwell filters in the presence (+) or absence (−) of fibronectin for 4 h. Data are presented as mean ± SEM of three independent experiments. * *p* (8 µm) = 0.0240; * *p* (3 µm) = 0.0371. (**C**) Representative 3D images of an intravascular (marked by a white contour) or a protrusive tumor cell crossing the CD31-labeled lung vasculature, along with the percentage of E0771 shControl and shLmna cells present in a volume of 5 × 10^9^ µm^3^ of the left lung lobe (3 h after retro-orbital injection) counted using Imaris software (*n* = 40 cells). Data are represented as mean ± SEM of two independent experiments. Scale bar, 100 µm. (**D**) In vitro cell growth of E0771 shControl or shLmna cells cultured replacing growth medium every 24 h. Data are represented as mean ± SEM of three independent experiments. (**E**) Growth arrest induced by etoposide at different concentrations on E0771shControl and shLmna cells after treatment for 72 h. Data are represented as mean ± SEM of three independent experiments. (**F**) Spheroid diameter measured on days 1, 3 and 6 after seeding (*n* = 50). Data are represented as mean ± SEM of two independent experiments. * *p* (0.0152); **** *p* < 0.0001; ns: nonsignificant. (**G**) Number of CMTMR-labeled E0771 shControl and shLmna cells present in the lungs of recipient mice 3 days after retro-orbital injection and 7 days after tail vein injection (*n* = 3; *n* = 4, shControl on day 3). (**H**,**I**) Experimental lung metastasis of E0771 breast cancer cells. 20,000 (**H**) or 10,000 (**I**) E0771 shControl and shLmna cells were injected into the tail vein of recipient C57BL/6 mice. After 14 days (**H**) or 30 days (**H**), animals were euthanized and lungs harvested. The number of micrometastases present in an H&E-stained section of the left lung lobe was determined using CaseViewer software (*n* = 4; *n* = 5). ** *p* (0.0030); * *p* (0.0246); *ns*: nonsignificant. Student’s two-tailed unpaired *t* test (**B**,**D**–**I**), two-way ANOVA with Bonferroni’s post hoc test (**C**).

We next reasoned that reduced lamin A/C levels could differentially impact distinct types of cancer cells. We therefore addressed how reduced lamin A/C expression in bona fide breast cancer cells, the E0771 line, affects their squeezing, migration, epigenetics and growth properties both in vitro and in vivo. Downregulation of lamin A/C levels with conserved lamin B content (Figure 7A), introduced by distinct shRNA-expressing lentiviral vectors or by siRNA, dramatically facilitated haptotactic cancer cell squeezing through rigid pores in vitro (Figure 7B and Figure S10A,B), in agreement with our findings on B16F10 cells (Figure 1D,E). While the nuclear circularity index of control E0771 cells was higher than that of control B16F10 cells (Figure 1C and Figure S10C), the nuclei of these cells underwent significant increases in deformability, i.e., reduced circularity, upon lamin A/C downregulation (Figure S10C,D), reminiscent of the effect of lamin A/C downregulation on B16F10 nuclei. As observed with B16F10 cells, the transendothelial migration capacity of these cells in vitro and in vivo was not augmented by reduced lamin A/C expression (Figure 7C and Figure S11A,B). Although downregulated lamin A/C expression resulted in slightly reduced H3K9Me3 heterochromatin content (Figure S12A), it did not increase global RNA transcription rates (Figure S12B). The alterations in gene expression caused by lamin A/C downregulation in E0771 cells also did not overlap those associated with lamin A/C downregulation in B16F10 cells (Figure 4C, Figure S4 and Figure S12C,D). Furthermore, primary breast cancer growth in vitro was unaffected by lamin A/C downregulation (Figure 7D). DNA damage-induced growth arrest was also insensitive to downregulated lamin A/C expression (Figure 7E). Nevertheless, and reminiscent of the proliferation results of B16F10 cells, lamin A/C knockdown E0771 cells did not undergo appreciable apoptosis (Figure S13), but expanded more slowly in spheroids grown in 3D conditions compared to control (Figure 7F). The similar recovery of lamin A/C knockdown E0771 cells from recipient lungs three and seven days post injection (Figure 7G) also indicated that lamin A/C knockdown E0771 cells did not experience elevated apoptosis due to shear stress associated damages [49]. Lamin A/C knockdown E0771 cells failed to generate any metastatic lesions in the lungs 14 and 30 days post injection (Figure 7H,I). Thus, reduced nuclear content of lamin A/C does not alter breast cancer cell extravasation from lung vessels into the lung parenchyma, and dramatically compromises the metastatic potential of these breast cancer cells in this organ, reminiscent of our observations with lamin A/C knockdown B16F10 melanoma cells.

## 4. Discussion

The nucleus is the most bulky organelle in all cells and is protected by a mechanically stable network underlying the inner nuclear membrane termed the nuclear lamina [13,57]. The main mechanical obstacle for the extravasation of solid cancer cells across blood vessels in vivo is their stiff nuclei [4]. The nuclear lamina is thought to affect the shape and the mechanical properties of the nucleus, and hence to control cell squeezing through different barriers [13,16,58]. The lamina also plays an important role in the maintenance of the nuclear envelope integrity as well as in the organization of the nucleus as a whole [1,11]. The nuclear lamina is also connected to highly condensed chromatin regions (heterochromatin) and can affect chromatin conformation, and epigenetics [59]. Lamin A/C expression is reduced in several solid cancers but not others, and so the molecular basis of these changes and their direct link to cancer cell migration, survival and expansion have been under debate reflecting the complexity and diversity of tumor growth, migration and metastasis [29,60,61].

We chose to address these key standing questions using two prototypic BL/6 metastatic cell lines, B16F10 melanoma and E0771 breast carcinoma. Taking both in vitro and in vivo reductionist approaches in syngeneic immunocompetent mice, we have systematically assessed how knockdown of A-type lamins affects specific growth and migratory properties in different environmental conditions both in vitro and in vivo. Our in vitro findings of enhanced squeezing capabilities of the lamin A/C knockdown cells in the transwell assay are predictable and in full agreement with previous results on the critical role of lamin A/C in the ability of cells of different origins to squeeze through rigid confinements in vitro [13,16,58]. Unexpectedly, we found that nuclear deformability has no impact on the overall nuclear squeezing kinetics through endothelial junctions, indicating that endothelial barriers are highly permissive for nuclear passage and well adapted to accommodate the squeezing of cells with bulky and stiff nuclei. We postulate that the exceptionally slow rates of tumor transendothelial migration may provide the endothelial cytoskeleton with sufficient time to undergo remodeling including activation of contractility machineries to facilitate the squeezing of solid tumor cells though junctions or transcellular pores, regardless of the relative stiffness of their nuclei [16,62]. In order to validate these unexpected in vitro TEM results, we developed a new experimental lung metastasis model to assess in vivo the intrinsic ability of our lamin A/C suppressed tumor cells to squeeze through the lung vasculature. Tumor metastasis into lungs occurs nearly exclusively through the pulmonary capillaries [46], an extensive network of relatively impermeable capillaries considered to be poorly permeable compared with vessels targeted by metastatic cells at other organs like the bone marrow and liver [47]. By introducing a method to accurately measure the relative efficiency of cancer cell emigration through these vessels, we found that highly invasive cells like melanoma B16F10 crossed these barriers in vivo independently of their lamin A/C content and irrespectively of their intrinsic nuclear deformability properties. Although poorly invasive, the ability of E0771 cells to cross identical pulmonary vascular barriers was also insensitive to their intrinsic nucleus deformability and lamin A/C content. Our in vivo results were therefore fully consistent with the transmigratory properties of these cells determined in our in vitro transendothelial migration setups.

Thus, our results argue that the squeezing ability of a given tumor cell through nondegradable rigid pores towards a chemotactic or a haptotactic signal does not predict the physiological crossing potential of that cell across cellular barriers. Although not addressed in our study, it is possible that upon entering the interstitial space, the ability of lamin A/C rich tumor cells to enzymatically degrade glycoprotein and proteoglycan components of collagenous barriers [63] is more critical for their interstitial motility than the mechanical restrictions imposed by the stiffness of their lamin A/C rich nuclei. It is also possible that lamin A/C-regulated crosstalks between the nucleus and MT1-MMP promote a digest-on-demand program in cells squeezing through constricted collagenous spaces [20]. Optimal connections of lamin A/C with the cell cytoskeleton (e.g., via the linker of nucleoskeleton and cytoskeleton (LINC) complex) can be, in fact, essential for optimal proteolysis of ECM barriers at the front of both the melanoma and breast carcinoma cells studied by us.

Previous observations showed that depletion of lamin A/C in lung and breast cancer cells, as well as fibrosarcoma cells, significantly increased the likelihood of transient nuclear envelope rupture events and cell death especially when cells were forced to migrate through very tight and rigid barriers [15]. Imaging of HT1080 fibrosarcoma cells invading the collagen-rich mouse dermis in live tumors after orthotopic implantation confirmed that migration-induced nuclear envelope rupture occurs in vivo, particularly during cell division [64] and in individually disseminating cells [15]. Nuclear envelope rupture is less prevalent, however, in cells moving as multicellular collective strands [15] or on 2D surfaces [61]. It can therefore be predicted that the nuclei of both lamin A/C knockdown B16F10 cells and E0771 cells reaching the lungs as individual cells would be sensitive to the lung microenvironment. This sensitivity is unlikely, however, to result of lower resistance of lamin A/C knockdown cells to shear stress induced damages [49] because the accumulation of these cells inside the lung vasculature determined 3 h post injection and the retention of these cells during the first three days after injection were not reduced for both lamin A/C deficient B16 and E0771 cells. Nevertheless, in correlation with their growth disadvantage inside the lung parenchyma, lamin A/C knockdown melanoma and breast cancer cells exhibited markedly reduced growth inside spheroid assemblies. It is therefore possible that once reaching the lungs and extravasating from blood vessels into specific compartments of the lung parenchyma, lamin A/C knockdown cells initially grow without any defect. Later on, these cells fail to expand in spheroids and this growth deficiency results in slower growth of metastatic lesions. At this point, we cannot conclusively determine, if the failure to generate metastatic lesions in the lungs reflects mere cell-intrinsic defects of tumor cell growth within spheroids, or also cell-extrinsic growth defects, resulting from compromised cancer cell communications with immune and non-immune cells accumulated around the metastatic lesions [65].

The contribution of high lamin A/C content to chromatin flexibility and DNA stability is still disputed. High lamin A/C content protects the nuclei from rupture and cell apoptosis occurring under extreme mechanical stress such as squeezing through rigid confinements [14,15] and also reduces the frequency of spontaneous aneuploidy [24] and DNA damage [66]. We did not find a direct link between lamin A/C deficiency and these cellular processes. Collectively, our results predict that lamin A/C deficiency does not provide any migratory advantages to cancer cells reaching the lung vasculature but reduces their metastatic potential in the lungs at a post extravasation step, possibly at the level of proliferation inside spheroids. The precise molecular basis for this lamin A/C related growth deficiency and its relevance to the metastatic spread of these cells in other organs awaits future elucidation.

## 5. Conclusions

In our study, we demonstrated that lamin A/C deficiency in different types of cancer cells slows down cell growth both in vitro and in vivo and restricts lung metastasis. This deficiency, as reported for other cellular systems, renders the nuclei of the various tumor cells deformable, and promotes cancer cell crossing through small rigid barriers. Nevertheless, it does not elevate the transendothelial capacity of these cells in vitro or in vivo. In conclusion, metastatic ability of two prototypes of circulating cancer cells is not elevated by reduced A-type lamins, as previously suggested, although we cannot extrapolate our findings to other cancer models. Similar systematic approaches should be taken to extend our observations to other types of cancers and cancer lines.

## Data Availability

Sequencing data have been deposited in NCBI’s Gene Expression Omnibus and are accessible through GEO Series accession number GSE151176.

## Supplementary Materials

The following are available online at https://www.mdpi.com/article/10.3390/cancers13102383/s1, Supplementary Material and Methods and key resources table. Figure S1: Clinical outcomes of elevated *LMNA* mRNA expression; Figure S2: Downregulation of lamin A/C introduced by a second shRNA (shLmna-2) or by siRNA increases B16F10 melanoma cells squeezing through small rigid pores; Figure S3: Quantification of cancer cells accumulation in lungs; Figure S4: Lamin A/C downregulation alters gene transcription; Figure S5: DNA damage-induced growth arrest and senescence of cancer cells induced by etoposide treatment in vitro; Figure S6: Lamin A/C downregulation does not alter R-loop (S9.6) formation in B16F10 cells; Figure S7: Melanoma proliferation in spheroids is reduced by lamin A/C downregulation introduced by a second shRNA; Figure S8: Proliferation of lamin A/C knockdown B16F10 cells in 3D spheroids is reduced also in the presence of serum enriched media as well as in hypoxic conditions; Figure S9: Apoptotic fraction of B16F10 cells isolated from recipient lungs on day 7; Figure S10: Downregulation of lamin A/C increases E0771 breast carcinoma cells squeezing through small rigid pores and alters nuclear shape; Figure S11: In vitro and in vivo breast carcinoma crossing of endothelial barriers is not enhanced by lamin A/C downregulation; Figure S12: Lamin A/C downregulation reduces heterochromatin content and alters gene transcription; Figure S13: The fraction of apoptotic cells in spheroids of either E0771 shControl or shLmna cells on day 6; Figure S14: Original information about the western blot. Table S1: DeSeq2 analysis output and gene expression levels. Video S1: Transendothelial migration of B16F10 murine melanoma; Video S2: Migratory categories of B16F10 cells over a bEnd.3 endothelium; Video S3: Transendothelial migration of B16F10 shControl vs shLmna cells; Video S4: Nuclear deformability of B16F10 shControl vs shLmna cells; Video S5: Light sheet microscopy of tumor cells, bronchial structures and lung vasculature; Video S6: Example of an intravascular B16F10 cell; Video S7: Example of a protruding B16F10 cell; Video S8: Example of an extravascular B16F10 cell; Video S9: Nuclear deformability of E0771 shControl or shLmna cells.
