# Supplementary material for: Reduced Lamin A/C Does Not Facilitate Cancer Cell Transendothelial Migration but Compromises Lung Metastasis
Source: Cancers (Basel). 2021 May 14;13(10):2383. doi: 10.3390/cancers13102383 (PMC8157058; doi:10.3390/cancers13102383)
Supplement: Supplementary file 1 [file cancers-13-02383-s001.zip › Supplementary Materials v2.pdf]

Supplementary

Reduced Lamin A/C Does Not Facilitate Cancer Cell Transendothelial Migration but Compromises Lung Metastasis

Francesco Roncato, Ofer Regev, Sara W. Feigelson, Sandeep Kumar Yadav, Lukasz Kaczmarczyk, Nehora Levi, Diana Drago-Garcia, Samuel Ovadia, Marina Kizner, Yoseph Addadi, João C. Sabino, Yossi Ovadya, Sérgio F. de Almeida, Ester Feldmesser, Gabi Gerlitz and Ronen Alon

Supplementary Material and Methods

Transient siRNA Transfection

B16F10 cells (1 × 10^5) were transfected with siRNA (20 nM) using Lipofectamine™ RNAiMAX (Thermo Fisher Scientific) following the manufacturer's instructions. 72 h after transfection, tumor cells were either lysed to quantify the mean knockdown protein levels or utilized in experimental assays. Lamin A/C siRNA (siLmna) sequence used in this study was described in [1].

Western Blotting

Cells were grown as described above, washed twice with ice-cold PBS, scraped into lysis buffer (25 mM Tris pH 7.5, 1 mM EDTA, 0.5 mM EGTA, 150 mM NaCl, 1% NP-40, 0.2% SDS, 2 mM Na_3VO_4, 1 mM NaF, 10 mM Nappi, 80 mM b-glycerol phosphate and a protease inhibitor tablet), and kept on ice for 30 min with occasional vortexing. Thereafter, lysates were centrifuged at 14,000 × g for 15 min at 4 °C. The supernatant was collected and protein concentration was determined by BCA protein assay (Thermo Fisher Scientific). The protein suspension was separated by gel electrophoresis followed by transfer to nitrocellulose membranes, and blocking with non-fat milk (5% in PBS-T) for 1 h at RT. Immunoblotting was performed overnight at 4°C according to the manufacturers' guidelines. Antibody binding to membrane blots was detected using horseradish peroxidase conjugated secondary antibodies for 1 h at RT, followed by development with a chemiluminescence substrate (Thermo Fisher Scientific). Chemiluminescence was detected using the ChemiDoc MP (Bio-Rad Laboratories) imaging system.

Immunofluorescence Staining

B16F10 or E0771 cells (1.5 × 10^4) were seeded into a µ-Slide VI0.4 ibiTreat (ibidi) pre-coated with fibronectin (10 µg/mL in PBS) for 30 min at 37 °C. The next day cells were rinsed with ice-cold PBS and fixed with paraformaldehyde (4% in PBS) for 15 min at RT followed by permeabilization with Triton X-100 (0.25% in PBS) for 15 min at RT and blocking with goat serum (10% in PBS) for 20 min at 37 °C.

The cells were then incubated with anti-lamin A/C antibody (1:100) for 1 h at RT, washed with ice-cold PBS three times and incubated with an Alexa Fluor 488 conjugated secondary antibody (1:200) for 1 h at RT. Cells were imaged using an IX83 inverted microscope (described above) equipped with an UPlanFLN 40X 0.75 Ph2 ∞/0.17/FN 26.5 objective (Olympus), 49002-ET-EGFP (FITC/Cy2) filter set (Chroma). Alternatively, cells were fixed with paraformaldehyde (3% in PBS) at RT for 5 min followed by fixation in methanol at −20 °C for another 5 min. H3K9me3 was detected with rabbit monoclonal anti H3K9me3 and DNA was stained with Hoechst 33258. Immunostaining images were collected using an Olympus IX81 fluorescent microscope equipped with a coolSNAP HQ2 CCD camera (Photometrics).

Run-on Transcription Assay (5-Ethynyl Uridine (EU) Labeling of Cultured Cells)

B16F10 or E0771 (either shControl or shLmna expressing) cells were grown on serum coated glass coverslips. 1 mM EU was added to the culture medium and cells were kept for 1 h at 37 °C in 5% CO_2. Cells were then washed with PBS and fixed with 3.7% formaldehyde for 15 min at RT. The fixative was removed and cells were washed twice with PBS, followed by permeabilization with 0.5% Triton X-100 for 15 min at RT. Cells were washed twice with PBS and incorporated EU was detected by click chemistry using a fluorescent azide following the manufacturer's guidelines of Click-iT RNA Imaging Kit (Thermo Fisher Scientific). Following the Click-iT reaction (30 min at RT in the dark), cells were rinsed twice with a rinse buffer. Nuclear staining was performed with Hoechst 33342. Cells were imaged using a Zeiss LSM 710 point scanning confocal microscope with stacking acquisition and generation of maximum intensity projection images. Nucleoplasmic fluorescence was quantified using Fiji.

Bulk MARS-seq Protocol and Sequencing

RNA was isolated from 10,000 cells from each cell line using Dynabeads® mRNA Direct Kit (Thermo Fisher Scientific). Libraries for RNA-seq were prepared using a modified version of TranSeq, as described [2]. Briefly, RNA was reversed transcribed with MARS-seq barcoded RT primers in a 10 μl volume with the Affinity Script kit (Agilent). Reverse transcription was analyzed by qRT-PCR and samples with a similar CT were pooled (up to eight samples per pool). Each pool was treated with Exonuclease I (NEB) for 30 min at 37 °C and cleaned by 1.2× volumes of SPRI beads (Beckman Coulter). Next, the cDNA was converted to double-stranded DNA with a second strand synthesis kit (NEB) in a 20 mL reaction, incubating for 2 h at 16 °C. The product was purified with 1.4× volumes of SPRI beads, eluted in 8 μl and in vitro transcribed (with the beads) at 37 °C overnight for linear amplification using the T7 High Yield RNA polymerase IVT kit (NEB). Following IVT, the DNA template was removed with Turbo DNase I (Ambion) 15 min at 37 °C and the

amplified RNA (aRNA) purified with 1.2 volumes of SPRI beads. The aRNA was fragmented by incubating 3 min at 70 °C in Zn^{2+} RNA fragmentation reagents (Ambion) and purified with 2× volumes of SPRI beads. The aRNA was ligated to the MARS-seq ligation adapter with T4 RNA Ligase I (NEB). The reaction was incubated at 22 °C for 2 h. After 1.5× SPRI cleanup, the ligated product was reverse transcribed using Affinity Script RT enzyme (Agilent) and a primer complementary to the ligated adapter. The reaction was incubated for 2 min at 42 °C, 45 min at 50 °C, and 5 min at 85 °C. The cDNA was purified with 1.5× volumes of SPRI beads. The library was completed and amplified through a nested PCR reaction with 0.5 mM of P5_Rd1 and P7_Rd2 primers and PCR ready mix (Kappa Biosystems). The amplified pooled library was purified with 0.7× volumes of SPRI beads to remove primer leftovers. Library concentration was measured with a Qubit fluorometer (Life Technologies) and mean molecule size was determined with a 2200 TapeStation instrument. RNA-seq libraries were sequenced using the Illumina NextSeq® 500 High Output v2 Kit (75 cycles).

Bioinformatics Analysis

Samples were demultiplexed using the barcode present in the R2 read. The analysis was performed using the UTAP pipeline [3]. In brief, UMI sequences present in the R2 read were inserted in the read name of R1 sequence file using a python script. Cutadapt was used to trim low quality, poly A and adapter sequences [4], (parameters: -a AGATCGGAAGAGCACACGTCTGAACTCCAGTCAC -a "A{10}" –times 2 -u 3 -u -3 -q 20 -m 25). Sequences were mapped to the UCSC mm10 mouse genome using STAR [5] v2.4.2a (parameters: –alignEndsType EndToEnd, –outFilterMismatchNoverLmax 0.05, –twopassMode Basic, –alignSoftClipAtReferenceEnds No). The pipeline quantified the 3′ of RefSeq annotated genes (1000 bases upstream of the 3′ end and 100 bases downstream) using HTSeq count [6] and a modified Refseq gtf file (downloaded from igenomes UCSC). UMI information was integrated into the BAM files as tags, using a python script. UMI counting was performed after marking duplicates (in-house script) using a modified HTSeq-count. DESeq2 7 was used for normalization and detection of differentially expressed genes. Raw p-values were adjusted for multiple testing using the procedure of Benjamini and Hochberg. Genes were considered to be differentially expressed if their mean normalized expression was greater than 5, the absolute value of the log2FoldChange was greater than 1, and the adjusted p-value was less than 0.05. Batch effects were removed from the read counts using the ComBat function from the sva R package [8]. The normalized, batch corrected and log2 transformed read counts outputted by ComBat were used to draw plots. K-means clustering of differentially expressed genes was performed using the Partek® Genomics Suite® software, version 6.6 (Partek Inc., St. Louis, MO, USA). Functional analysis of the differentially expressed genes was performed using https://metascape.org/gp/index.html#/main/step1,

Metascape [9]. Significantly enriched GO terms for up and downregulated genes were extracted from this analysis.

Senescence-Associated-β-Galactosidase (SA-β-Gal) Activity

For SA-β-gal activity assay, B16F10 cells (shControl or shLmna) were treated with 5 μM etoposide for 72 h, washed and left in regular culture medium (containing 2.5 μg/mL of puromycin) for an additional 120 h. Cells were then fixed with 0.5% glutaraldehyde in PBS for 15 min, washed twice with PBS supplemented with 1 mM $MgCl_2$ (pH 5.5) and stained with PBS containing 1 mM $MgCl_2$ (pH 5.5) and supplemented with 1 mg/mL X-gal, 5 mM $K_3Fe[CN]_6$ and 5 mM $K_4Fe[CN]_6$ for 5 h at 37 °C in the dark, as described [10]. Cells were washed with warm PBS, fixed with 4% paraformaldehyde and imaged using a SZX16 stereo microscope (Olympus) equipped with SDF PLAPO 1XPF objective (Olympus) set at 10X magnification. DP73 camera (Olympus).

R-loops Quantification

B16F10 cells were grown on sterile coverslips and R-loops were detected with the S9.6 antibody following cell fixation and permeabilization with 100% ice-cold methanol and acetone for 10 min and 1 min on ice, respectively. Incubation with primary antibodies was followed by incubation with Dy488-secondary antibody (Bethyl). Nuclei were stained using DAPI and coverslips were assembled in Vectashield Mounting Medium (H-1000, Vector Laboratories). All the washing steps were done with PBS containing 0.05% (*v*/*v*) Tween 20. Images were acquired using Confocal Laser Point-Scanning Microscope Zeiss LSM 710. Fluorescence intensity of the nucleoplasmic staining was assessed using ImageJ.

Key Resources Table.

Reagent Type (Species) or Resource	Designation	Source or Reference	Identifiers	Additional Information
Strain, strain background (*Mus musculus*)	C57BL/6	The Jackson Laboratory		
Cell line (*Homo sapiens*)	HEK293T	ATCC	ATCC CRL-3216	
Cell line (*Mus musculus*)	bEnd.3	Gift from Britta Engelhardt, Theodor Kocher Institute, University of Bern	ATCC CRL-2299	
Cell line (*Mus musculus*)	B16F10	Gift from Prof. Lea Eisenbach, Weizmann Institute of Science	ATCC CRL-6475	
Cell line (*Mus musculus*)	E0771	Gift from Dr. Ravid Straussman, Weizmann Institute of Science	CH3 BioSystems Cat# 94A001	
Cell line (Mus *musculus*)	LL/2 (LLC1)	Gift from Dr. Ravid Straussman, Weizmann Institute of Science	ATCC CRL-1642	
Antibody	anti-Lamin A/C (4C11)	Cell Signaling Technology	Cat# 4777, RRID:AB_10545756	
Antibody	anti-Lamin B1 [EPR8985(B)]	Abcam	Cat# ab133741, RRID:AB_2616597	
Antibody	anti-GAPDH (6C5)	Millipore	Cat# MAB374, RRID:AB_2107445	
Antibody	Alexa Fluor® 647 anti-CD31 (390)	BioLegend	Cat# 102416, RRID:AB_493410	
Antibody	anti-trimethyl Histone H3 (Lys9) (D4W1U)	Cell Signaling Technology	Cat# 13969, RRID:AB_2798355	
Antibody	anti-KMT1B/SUV39H2 [EPR18495]	Abcam	Cat# ab190870, RRID:AB_2827544	
Antibody	anti-trimethyl Histone H3 (Lys27) (18E9.1)	Millipore	Cat# 05-1951, RRID:AB_11211815	
Antibody	anti-Histone H3 (A3S)	Millipore	Cat# 05-928, RRID:AB_492621	
Antibody	anti-ESET/SETDB1 (D4M8R)	Cell Signaling Technology	Cat# 93212, RRID:AB_2800200	
Antibody	anti-alpha Tubulin (DM1A)	Thermo Fisher Scientific	Cat# 62204, RRID:AB_1965960	
Antibody	anti-DNA-RNA Hybrid (S9.6)	Millipore	Cat# MABE1095, RRID:AB_2861387	
Antibody	anti-Ki67	Abcam	Cat# ab16667, RRID:AB_302459	
Antibody	Alexa Fluor® 647 anti-rabbit IgG (H+L)	Jackson ImmunoResearch Labs	Cat# 711-605-152, RRID:AB_2492288	
Antibody	anti mouse IgG+IgM+IgA cross-adsorbed (H+L)	Bethyl	Cat# A90-244D2, RRID:AB_10683272	
Antibody	Alexa Fluor® 488 anti-mouse IgG (H+L)	Thermo Fisher Scientific	Cat# A-21202, RRID:AB_141607	
Antibody	Peroxidase-AffiniPure anti-mouse IgG (H+L)	Jackson ImmunoResearch Labs	Cat# 115-035-003, RRID:AB_10015289	

Antibody	Peroxidase-AffiniPure anti-rabbit IgG (H+L)	Jackson ImmunoResearch Labs	Cat# 111-035-003, RRID:AB_2313567	
Recombinant DNA reagent	MISSION® TRC2 pLKO.5-puro Empty Vector (shControl)	Sigma-Aldrich	Cat# SHC201	
Recombinant DNA reagent	MISSION® shRNA plasmid DNA Lamin A/C (shLmna)	Sigma-Aldrich	TRCN0000317672	
Recombinant DNA reagent	MISSION® shRNA plasmid DNA Lamin A/C (shLmna-2)	Sigma-Aldrich	TRCN0000089849	
Sequence-based reagent	Control siRNA (siControl)	Bioneer	Sense: UUCUCCGAACGUGUCACGUtt; anti-sense: ACGUGACACGUUCGGAGAAtt	[1]
Sequence-based reagent	Lamin A/C siRNA (siLmna)	Bioneer	Sense: GGCUUGUGGAGAUCGAUAAtt; anti-sense: UUAUCGAUCUCCACAAGCCgc	[1]
Sequence-based reagent	*Lmna* Primers	Integrated DNA Technologies	Forward: GGATGCTGAGAACAGGCTACA Reverse: CTCTCGCTGCTTCCCGTTATC	[11]
Sequence-based reagent	*Yap1* Primers	Integrated DNA Technologies	Forward: TACTGATGCAGGTACTGCGG Reverse: TCAGGGATCTCAAAGGAGGAC	[11]
Sequence-based reagent	*Gapdh* Primers	Integrated DNA Technologies	Forward: AGGTCGGTGTGAACGGATTTG Reverse: TGTAGACCATGTAGTTGAGGTCA	[11]
Sequence-based reagent	*Birc5* Primers	Integrated DNA Technologies	Forward: TGACAACCCGATAGAGGAGCATA Reverse: TTCTTCCATCTGCTTCTTGACAGT	
Sequence-based reagent	*Cyr61* Primers	Integrated DNA Technologies	Forward: CGGAGGTGGAGTTAACGAGAAA Reverse: AAGACAGGAAGCCTCTTCAGTGAG	
Peptide, recombinant protein	Fibronectin	Sigma-Aldrich	Cat# F1141	
Peptide, recombinant protein	Gelatin from bovine skin	Sigma-Aldrich	Cat# G9391	
Peptide, recombinant protein	Collagenase, Type 4	Worthington Biochemical	Cat# LS004188	
Peptide, recombinant protein	DNase I, grade II	Roche	Cat# 10104159001	
Peptide, recombinant protein	Recombinant murine HGF	PeproTech	Cat# 315-23	
Peptide, recombinant protein	Recombinant murine EGF	PeproTech	Cat# 315-09	
Peptide, recombinant protein	Recombinant murine $VEGF_{165}$ (VEGF-A)	PeproTech	Cat# 450-32	
Peptide, recombinant protein	Recombinant murine SDF-1α (CXCL12)	PeproTech	Cat# 250-20A	

Peptide, recombinant protein	Fibronectin	Sigma-Aldrich	Cat# F1141
Chemical compound, drug	Etoposide	Sigma-Aldrich	Cat# E1383
Chemical compound, drug	Puromycin	Sigma-Aldrich	Cat# P8833
Chemical compound, drug	CellTracker™ Orange CMTMR Dye	Thermo Fisher Scientific	Cat# C2927
Chemical compound, drug	Hoechst 33342	Thermo Fisher Scientific	Cat# 62249
Chemical compound, drug	DAPI	Sigma-Aldrich	Cat# D9542
Chemical compound, drug	X-Gal	Sigma-Aldrich	Cat# 11680293001
Chemical compound, drug	Ethyl Cinnamate (ECi)	Acros Organics	Cat# 103-36-6
Chemical compound, drug	cOmplete™, Mini, EDTA-free Protease Inhibitor Cocktail	Roche	Cat# 4693159001
Chemical compound, drug	Poly(ethylene glycol) (PEG8000)	Sigma-Aldrich	Cat# 81268
Chemical compound, drug	Triton X-100	Sigma-Aldrich	Cat# X100
Commercial assay or kit	Click-iT™ RNA Alexa Fluor™ 594 Imaging Kit	Thermo Fisher Scientific	Cat# C10330Cat# 4693159001
Commercial assay or kit	APC Annexin V Apoptosis Detection Kit with PI	BioLegend	Cat# 640932
Commercial assay or kit	Dynabeads™ mRNA DIRECT™ Purification	Thermo Fisher Scientific	Cat# 61011
Commercial assay or kit	Total RNA Mini Plus	A&A Biotechnology	Cat. #036-25
Commercial assay or kit	qScriptTM cDNA Synthesis Kit	Quantabio	Cat. #95047
Commercial assay or kit	SuperSignal™ West Pico PLUS Chemiluminescent Substrate	Thermo Fisher Scientific	Cat# 34577
Software, algorithm	cellSens (v1.16)	Olympus	https://www.olympus-lifescience.com
Software, algorithm	Imaris (v9.5.1)	Oxford Instruments	https://imaris.oxinst.com/packages
Software, algorithm	CaseViewer 2.3	3DHISTECH	https://www.3dhistech.com/caseviewer
Software, algorithm	Fiji	SciJava	https://fiji.sc
Software, algorithm	CytExpert	Beckman Coulter	www.beckman.com
Software, algorithm	Flowjo (v10.6.1)	FlowJo	https://flowjo.com
Software, algorithm	Image Lab (v6.0.1)	Bio-Rad Laboratories	https://www.bio-rad.com
Software, algorithm	GraphPad Prism 6	GraphPad Software	https://www.graphpad.com
Software, algorithm	R	R Development Core Team	https://www.r-project.org
Software, algorithm	UTAP pipeline	[3]	https://utap.readthedocs.io
Other	Lipofectamine™ 2000	Thermo Fisher Scientific	Cat# 11668
Other	Lipofectamine™ RNAiMAX	Thermo Fisher Scientific	Cat# 3778075
Other	Matrigel® Matrix	Corning	Cat# 356234
Other	Poly-L-lysine solution	Sigma-Aldrich	Cat# P4832
Other	Agarose, low gelling temperature	Sigma-Aldrich	Cat# 9045
Other	Cell Dissociation Solution	Biological Industries	Cat# 03-071-1B
Other	Red Blood Cell Lysing Buffer Hybri-Max™	Sigma-Aldrich	Cat# R7757
Other	DMEM, high glucose	Thermo Fisher Scientific	Cat# 41965

Supplementary Figures

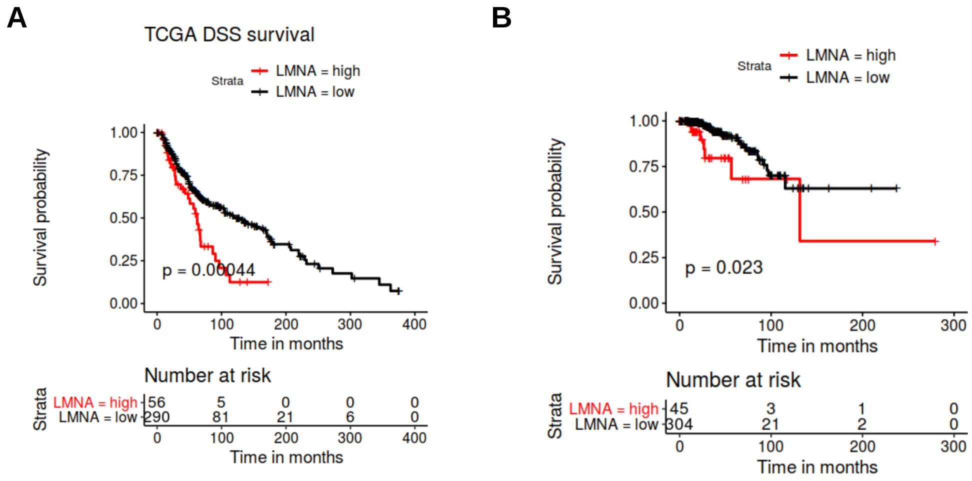

Figure S1. Clinical outcomes of elevated *LMNA* mRNA expression. Kaplan-Meier curves for Disease Specific Survival according to the levels of *LMNA* mRNA expression in patients from the TCGA cohort diagnosed with (**A**) metastatic skin cutaneous melanoma or (**B**) invasive breast carcinoma (Luminal B subtype). The number of patients (number at risk) is displayed below each curve with intervals of 100 months.

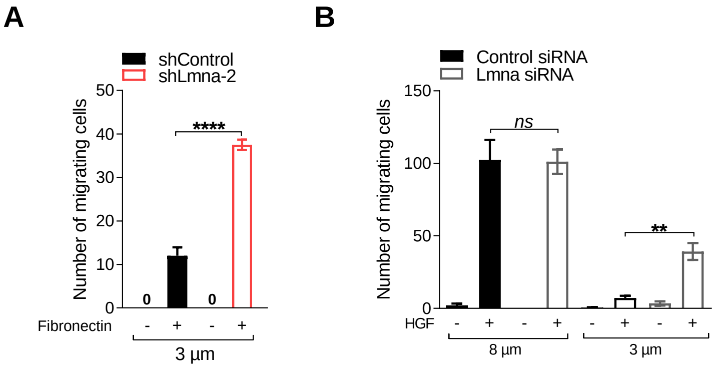

Figure S2. Downregulation of lamin A/C introduced by a second shRNA (shLmna-2) or by siRNA increases B16F10 melanoma cells squeezing through small rigid pores. (**A**) Haptotactic migration (4 h) of B16F10 shControl and shLmna-2 through 3 µm pore transwell filters coated (+) or uncoated (−) with fibronectin (1.5 µg/mL), quantified after 4 h. Data are represented as mean ± SEM of two independent experiments. **** $p < 0.0001$. (**B**) Migration of B16F10 siControl or siLmna cells (72 h after transfection) towards HGF (50 ng/mL) through 8 or 3 µm pore transwell filters quantified after 4 h. ** p (0.0076); ns: nonsignificant. Data are represented as mean ± SEM of two independent experiments.

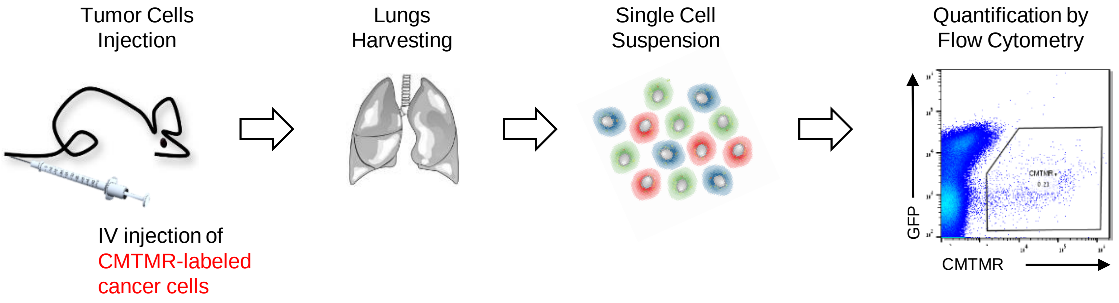

Figure S3. Quantification of cancer cells accumulation in lungs. Scheme depicting the experimental pipeline necessary to quantify single cell accumulation in lungs of recipient mice. Cancer cells are labeled with CMTMR orange cell tracker for 30 min and IV injected into recipient mice. After 3 hours, 3 or 7 days, the mice are euthanized, their lungs harvested, minced and digested into a single cell suspension that is subsequently analyzed by flow cytometry.

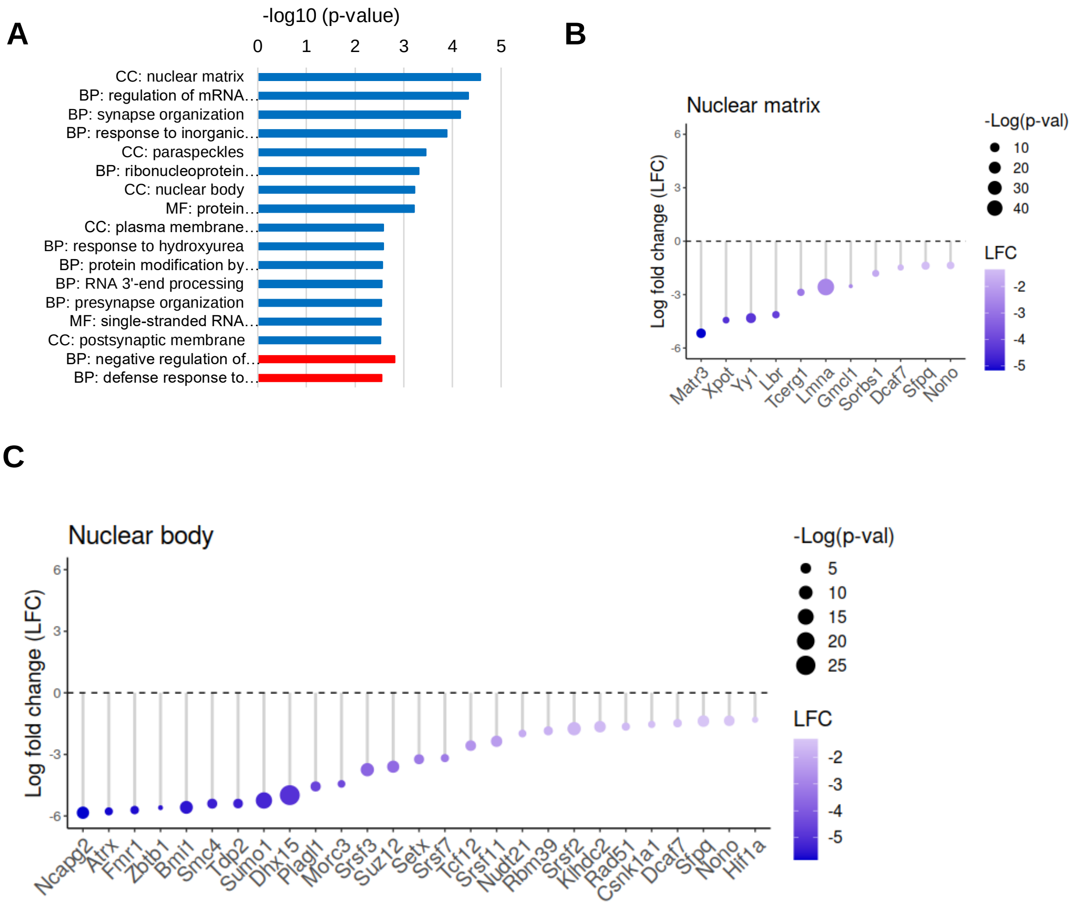

Figure S4. Lamin A/C downregulation alters gene transcription. (**A**) Gene ontology (GO) enrichment analysis of the top differentially downregulated (blue) and upregulated (red) genes in B16F10 shLmna cells. Biological Process (BP), Molecular Function (MF) and Cellular Component (CC). The full list of differentially expressed genes available in Table S1. (**B**,**C**) List of differentially expressed genes for the enriched gene ontology (GO) terms: nuclear matrix (B) and nuclear body (C); size of the dots represents statistical significance (adjusted *p* value).

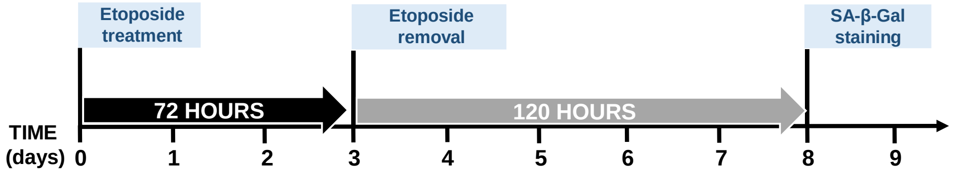

Figure S5. DNA damage-induced growth arrest and senescence of cancer cells induced by etoposide treatment in vitro. Cancer cells were exposed to 5 μM etoposide for 72 h. The compound was subsequently removed and the cells were cultured with regular growth medium for additional 120 h. Cells were then fixed in 0.5% glutaraldehyde for 15 min and stained for senescence associated β-Galactosidase as described in [10].

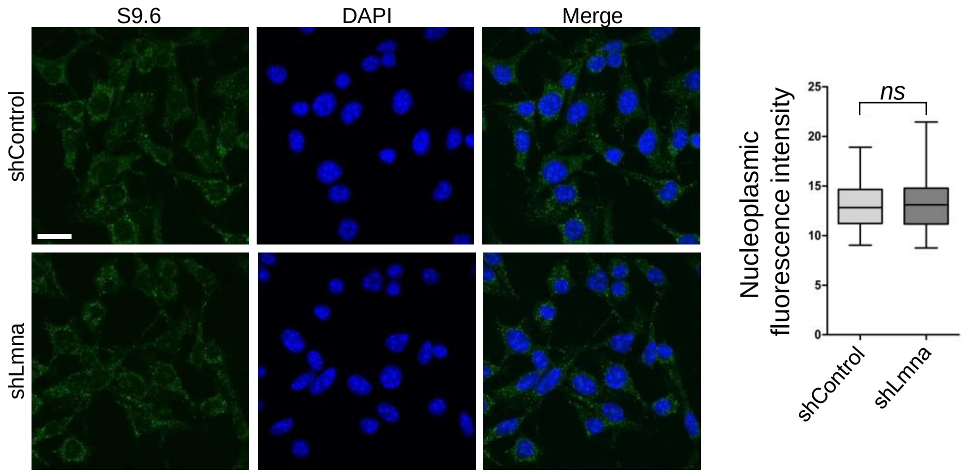

Figure S6. Lamin A/C downregulation does not alter R-loop (S9.6) formation in B16F10 cells. R-loop mapping via S9.6-based anti-DNA:RNA hybrid antibody. Nucleoplasmic fluorescence was quantified using FIJi software ($n = 90$). Data are represented as mean ± SEM of three independent experiments. Mann-Whitney two-tailed U test. Scale bar, 25 μm.

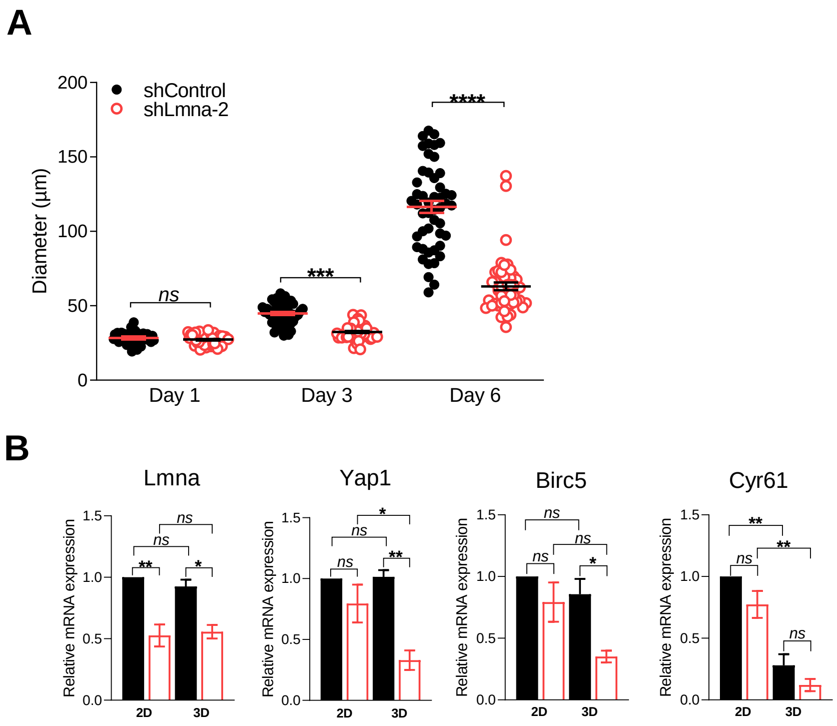

Figure S7. Melanoma proliferation in spheroids is reduced by lamin A/C downregulation introduced by a second shRNA (shLmna-2). (**A**) Soft agar colony formation assay. B16F10 shControl or shLmna-2 cells were embedded in soft agar and their growth was monitored for 6 days. Spheroid diameter was measured on days 1, 3, and 6 after seeding ($n = 50$). *** $p = 0.004$; **** $p < 0.0001$; ns: nonsignificant. Data are represented as mean ± SEM of two independent experiments. (**B**) Relative levels of *Lmna*, *Yap1*, and *Yap1*-related genes assessed by qRT-PCR for control and lamin A/C knockdown B16F10 cells grown on 2D or within 3D spheroids isolated on day 6 post seeding. Values are relative to shControl grown in 2D. ** $p = 0.0036$. Data are represented as mean ± SEM of three independent experiments. * $p < 0.05$; ** $p < 0.005$; *** $p < 0.0005$; **** $p < 0.0001$. Student's two-tailed unpaired t test (**A**) or one-way ANOVA with Tukey's post hoc test (**B**).

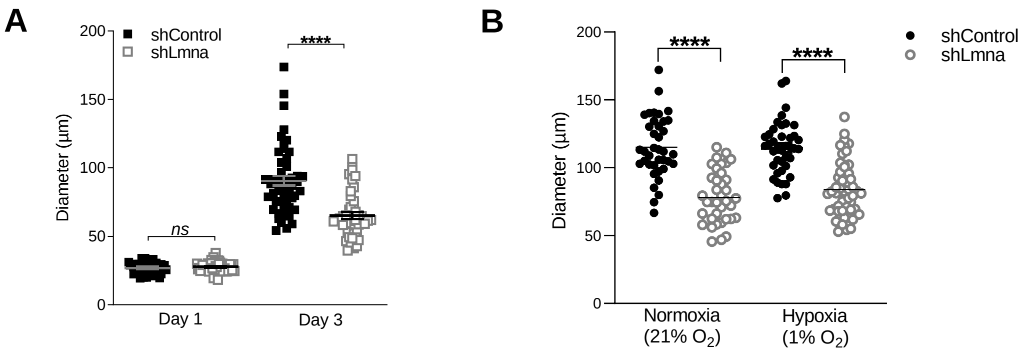

Figure S8. Proliferation of lamin A/C knockdown B16F10 cells in 3D spheroids is reduced in the presence of serum enriched media as well as in hypoxic conditions. (**A**) The diameter of individual spheroids measured on days 1 and 3 grown in soft agar supplemented with 50% FBS ($n = 50$). Data are represented as mean ± SEM of two independent experiments. **** $p < 0.0001$; ns: nonsignificant. (**B**) The diameter of individual spheroids grown in 10% serum supplemented soft agar either in normoxic (21% O_2) or hypoxic (1% O_2) conditions for 6 days ($n = 50$). **** $p < 0.0001$. Student's two-tailed unpaired t test (**A**,**B**).

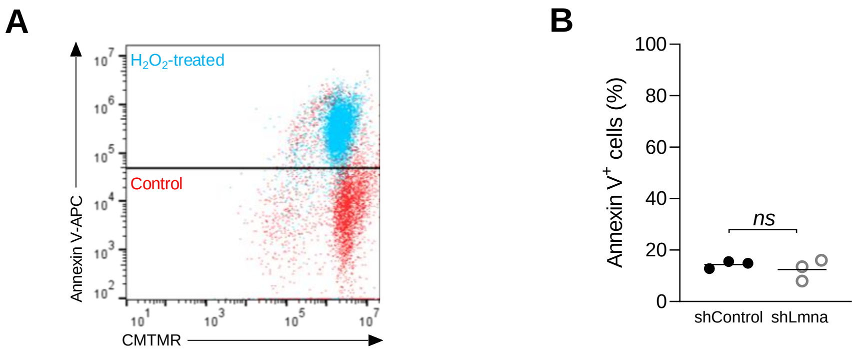

Figure S9. Apoptotic fraction of B16F10 cells isolated from recipient lungs on day 7 post injection. (**A**) In vitro readout of apoptosis. Representative flow cytometry dot plot of CMTMR-labeled B16F10 cells treated with 3% H_2O_2 or control solution for 15 min and subsequently stained for Annexin V. (**B**) A single cell suspension of recipient lungs was stained on day 7 post tail vein injection for Annexin V. CMTMR-labeled shControl or shLmna expressing B16F10 cells were gated as in Figure S3 (n = 3). ns: nonsignificant. Student's two-tailed unpaired t test (**B**).

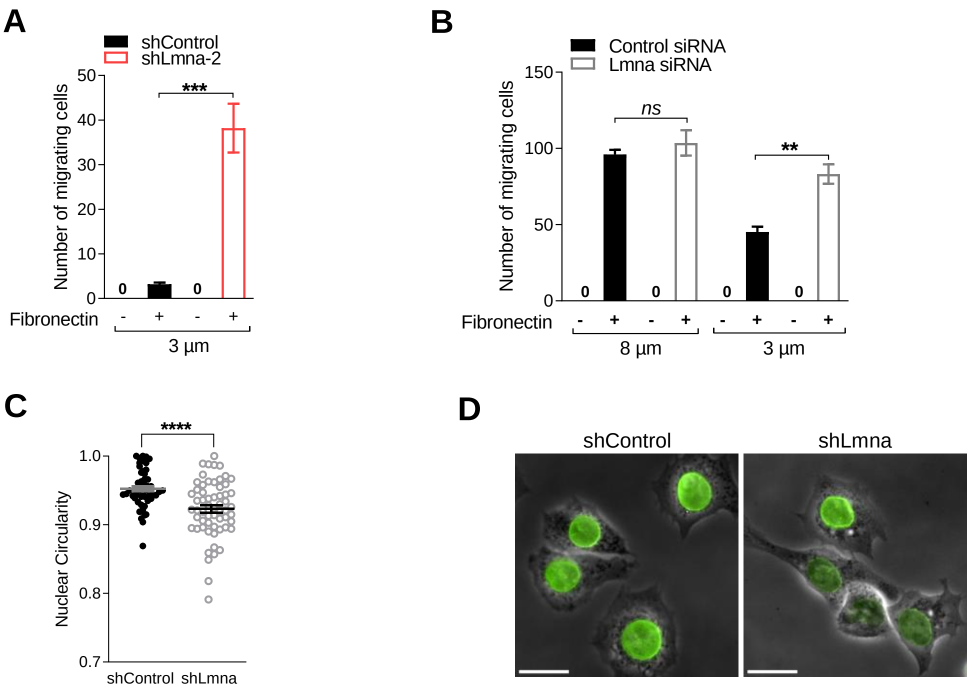

Figure S10. Downregulation of lamin A/C increases E0771 breast carcinoma cells squeezing through small rigid pores and alters nuclear shape. (**A**) Haptotactic migration of E0771 shControl or shLmna-2 cells through 3 µm pore transwell filters coated (+) or uncoated (−) with fibronectin (1.5 µg/mL), quantified after 4 h. Data are represented as mean ± SEM of two independent experiments. *** p (0.0002). (**B**) Haptotactic migration of E0771 siControl and siLmna (72 h post transfection), through 8 or 3 µm pore transwell filters coated (+) or uncoated (-) with fibronectin (1.5 µg/mL), quantified after 4 h. Data are represented as mean ± SEM of two independent experiments. ** p (0.0018); ns: nonsignificant. (**C**) Nuclear circularity of E0771 shControl or shLmna cells spread on a bEnd.3-derived basement membrane (n = 51, shControl; n = 60, shLmna). Data are represented as mean ± SEM. **** p <0.0001. (**D**) Representative immunostaining of lamin A/C (green),

superimposed on phase contrast images of E0771 shControl or shLmna cells. Scale bar, 20 µm. Student's two-tailed unpaired t test (**A**–**C**).

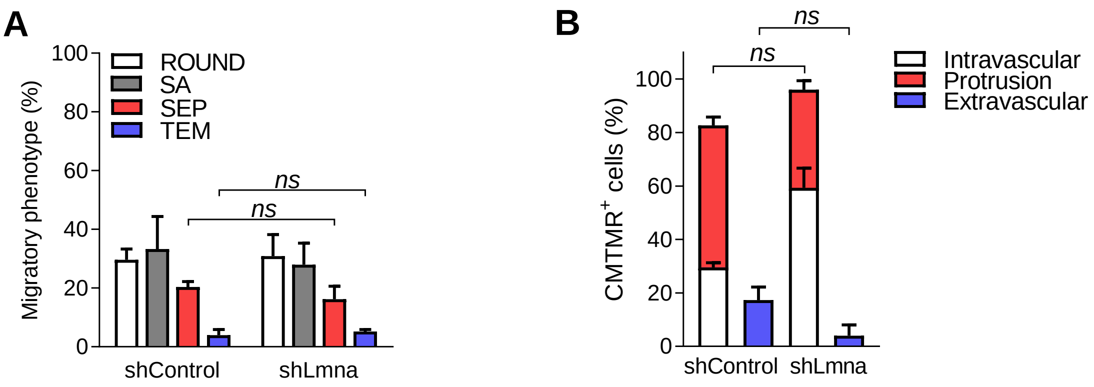

Figure S11. In vitro and in vivo breast carcinoma crossing of endothelial barriers is not facilitated by lamin A/C downregulation. (**A**) Migratory phenotypes of E0771 breast carcinoma cells TEM. Distinct tumor cell categories (referred to as migratory phenotypes) taken from time-lapse video microscopy segments of individual E0771 cells: round, spread above (SA), forming sub endothelial pseudopodia (SEP), and completing transendothelial migration (TEM) (n = 60). Data are represented as mean ± SEM of two independent experiments. (**B**) Percentage of E0771shControl and shLmna cells present in a volume of 5×10^9 μm^3 of the left lung lobe isolated 3 days after retro-orbital injection and counted with Imaris software (n = 40 cells). Data are represented as mean ± SEM of two independent experiments. ns: nonsignificant. Two-way ANOVA with Bonferroni's post hoc test (**A**,**B**).

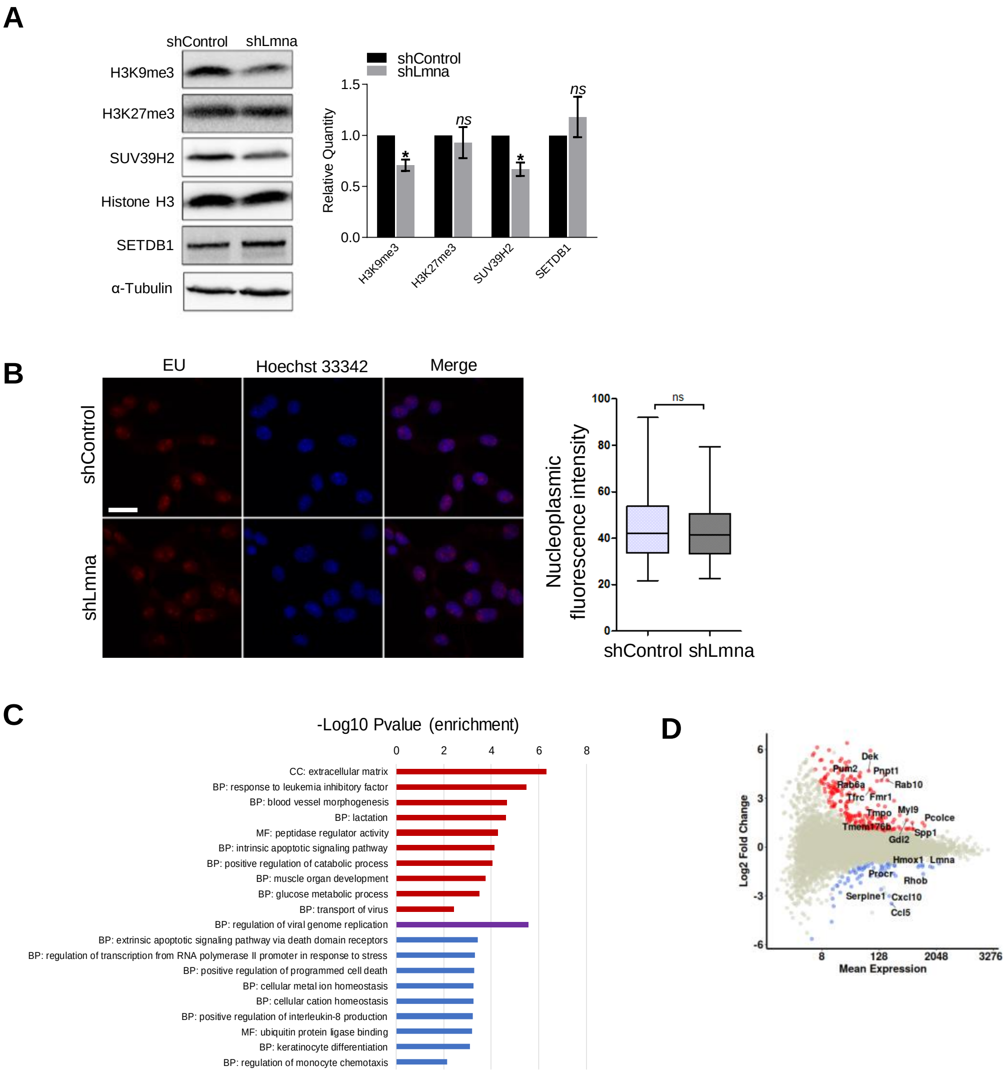

Figure S12. Lamin A/C downregulation reduces heterochromatin content and alters gene transcription. (**A**) Equal protein amounts from E0771shControl or shLmna cells, separated by SDS-PAGE and analyzed for the indicated proteins by Western blot analysis. The bar graph represents the mean levels of H3K9me3, H3K27me3 and SUV39H2 normalized to Histone H3 and of SETDB1 normalized to α-Tubulin ± SEM of at least four independent experiments. *$p < 0.05$; ns: nonsignificant. (**B**) Fluorescence microscopy imaging of 5-ethynyl uridine (EU) incorporation (red) and Hoechst 33342 (blue) in E0771 shControl and shLmna cells (n = 90). Data are represented as mean ±SEM of three independent experiments. Scale bar, 25 μm. (**C**) Gene ontology (GO) enrichment analysis of the top differentially downregulated (blue) and upregulated (red) genes in E0771 shLmna cells. Biological Process (BP), Molecular Function (MF) and Cellular Component (CC). (**D**) Log2FoldChange versus mean expression levels of differentially downregulated (blue), upregulated (red) and nondifferentially expressed genes (grey) are shown. The names of the top 20 most significantly expressed genes are indicated in the plot. Student's two-tailed unpaired t test (**A**), Mann–Whitney two-tailed U test (**B**).

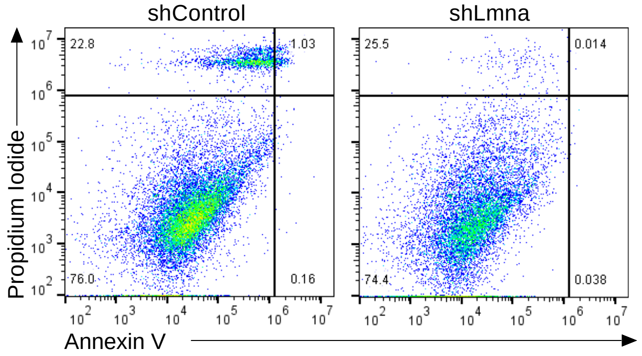

Figure S13. The fraction of apoptotic cells in spheroids of E0771 shControl or shLmna derived on day 6. Flow cytometry plots showing annexin V (X-axis) and propidium iodide (Y-axis) staining of either shControl or shLmna expressing E0771 cells extracted from 3D spheroids on day 6. The left lower quadrant indicates viable cells.

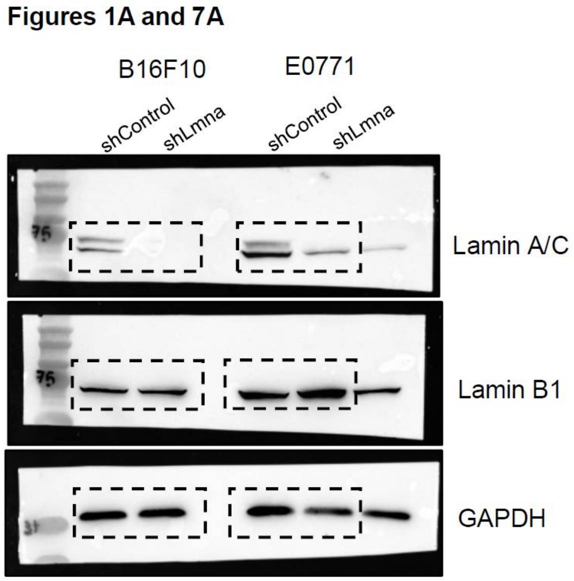

Figure S14. *cont.*

Figure 4B

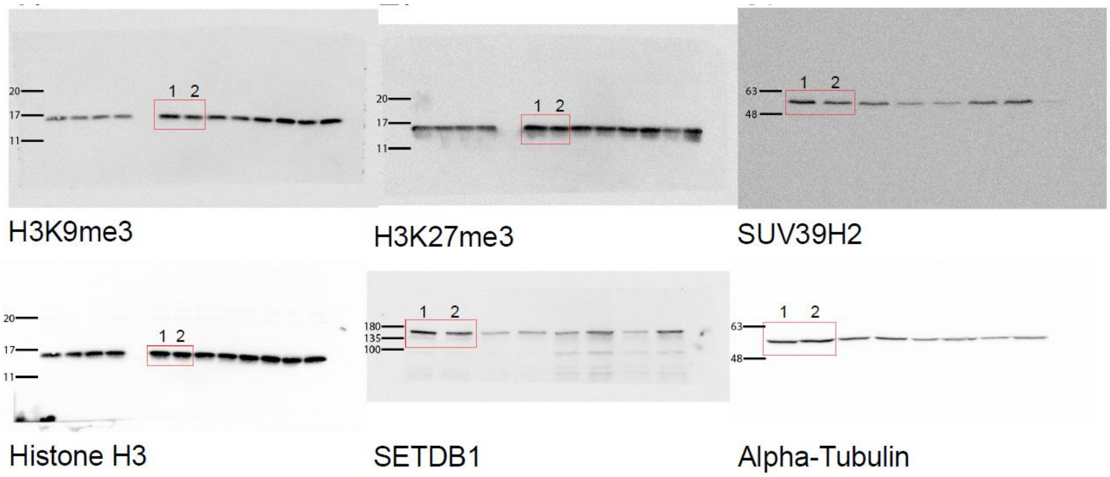

1 B16F10 shControl
2 B16F10 shLmna

Figure S11A

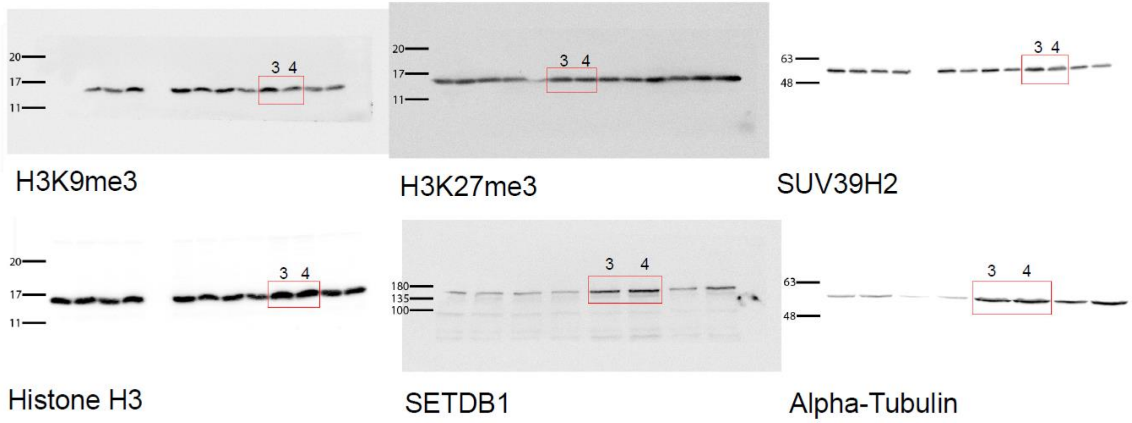

3 E0771 shControl
4 E0771 shLmna

Figure S14. Original information about the western blot.

Supplementary Video Legends

Video S1. Transendothelial migration of B16F10 murine melanoma. Time-lapse Video of a Hoechst-labeled B16F10 crossing a bEnd.3 endothelial monolayer. The contours of the tumor cell leading edges and nucleus are outlined in each image in yellow and red respectively. Elapsed time is designated as h:mm:ss. Scale bar, 20 μm.

Video S2. Migratory phenotypes of B16F10 cells over a bEnd.3 endothelium. Time-lapse Video divided in four quadrants in which different B16F10 cells display a unique phenotype when interacting with a bEnd.3 endothelial monolayer. Represented clockwise there are Round, Spread Above (SA), subendothelial pseudopodium (SEP) and transendothelial migration (TEM) phenotypes. Elapsed time is designated as h:mm:ss. Scale bar, 20 μm.

Video S3. Transendothelial migration of B16F10 shControl vs shLmna cells. Time-lapse Video depicting a Hoechst labeled B16F10 cell shControl (left) and shLmna (right) crossing a bEnd.3 endothelium. The contours of the tumor cell leading edges and nucleus are outlined in yellow and red respectively. Elapsed time is designated as h:mm:ss. Scale bar, 20 μm.

Video S4. Nuclear deformability and motility of B16F10 shControl vs shLmna cells. Time-lapse Video depicting the Hoechst-labeled nuclei (green) of B16F10 shControl (left) and shLmna (right) cells interacting with a bEnd.3-deposited basement membrane. Elapsed time is designated as h:mm:ss. Scale bar, 20 μm.

Video S5. Light sheet microscopy of tumor cells, bronchial structures and lung vasculature. Three-dimensional animated visualization of a section of murine lung lobe. CMTMR-labeled tumor cells (red) and autofluorescent bronchial structures (green) can be observed from seconds 0 to 21 (Video length). CD31-labeled lung vasculature (cyan) can be observed from seconds 22-40 (Video length).

Video S6. Example of an intravascular B16F10 cell. Three-dimensional animated visualization of a CMTMR-labeled B16F10 cell (red) located inside a CD31-labeled lung vasculature (cyan). Scale bar, 100 μm.

Video S7. Example of a protruding B16F10 cell. Three-dimensional animated visualization of a CMTMR-labeled B16F10 cell (red) protruding through the CD31-labeled lung vasculature (cyan). Scale bar, 100 μm.

Video S8. Example of an extravascular B16F10 cell. Three-dimensional animated visualization of a CMTMR-labeled B16F10 cell (red) located outside the CD31-labeled lung vasculature (cyan). Scale bar, 100 μm.

Video S9. Nuclear deformability and motility of E0771 shControl vs shLmna cells. Time-lapse video depicting the Hoechst-labeled nuclei (green) of shControl (left) and shLmna (right) expressing cells interacting with a bEnd.3-deposited basement membrane. Elapsed time is designated as h:mm:ss. Scale bar, 20 μm.

References

1. Roman, W.; Martins, J. P.; Carvalho, F. A.; Voituriez, R.; Abella, J. V. G.; Santos, N. C.; Cadot, B.; Way, M.; Gomes, E. R. Myofibril Contraction and Crosslinking Drive Nuclear Movement to the Periphery of Skeletal Muscle. *Nat. Cell Biol.* **2017**, *19*, 1189–1201.
2. Jaitin, D. A.; Kenigsberg, E.; Keren-Shaul, H.; Elefant, N.; Paul, F.; Zaretsky, I.; Mildner, A.; Cohen, N.; Jung, S.; Tanay, A.; Amit, I. Massively Parallel Single-Cell RNA-Seq for Marker-Free Decomposition of Tissues into Cell Types. *Science* **2014**, *343*, 776–779.
3. Kohen, R.; Barlev, J.; Hornung, G.; Stelzer, G.; Feldmesser, E.; Kogan, K.; Safran, M.; Leshkowitz, D. UTAP: User-Friendly Transcriptome Analysis Pipeline. *BMC Bioinformatics* **2019**, *20*. https://doi.org/10.1186/s12859-019-2728-2.
4. Martin, M. Cutadapt Removes Adapter Sequences from High-Throughput Sequencing Reads. *EMBnet.journal* **2011**, *17*, 10.
5. Dobin, A.; Davis, C. A.; Schlesinger, F.; Drenkow, J.; Zaleski, C.; Jha, S.; Batut, P.; Chaisson, M.; Gingeras,

T. R. STAR: Ultrafast Universal RNA-Seq Aligner. *Bioinformatics* **2013**, *29*, 15–21.
6. Anders, S.; Pyl, P. T.; Huber, W. HTSeq-A Python Framework to Work with High-Throughput Sequencing Data. *Bioinformatics* **2015**, *31*, 166–169.
7. Love, M. I.; Huber, W.; Anders, S. Moderated Estimation of Fold Change and Dispersion for RNA-Seq Data with DESeq2. *Genome Biol.* **2014**, *15*, 550.
8. Johnson, W. E.; Li, C.; Rabinovic, A. Adjusting Batch Effects in Microarray Expression Data Using Empirical Bayes Methods. *Biostatistics* **2007**, *8*, 118–127.
9. Zhou, Y.; Zhou, B.; Pache, L.; Chang, M.; Khodabakhshi, A. H.; Tanaseichuk, O.; Benner, C.; Chanda, S. K. Metascape Provides a Biologist-Oriented Resource for the Analysis of Systems-Level Datasets. *Nat. Commun.* **2019**, *10*, 1523.
10. Krizhanovsky, V.; Yon, M.; Dickins, R. A.; Hearn, S.; Simon, J.; Miething, C.; Yee, H.; Zender, L.; Lowe, S. W. Senescence of Activated Stellate Cells Limits Liver Fibrosis. *Cell* **2008**, *134*, 657–667.
11. Spandidos, A.; Wang, X.; Wang, H.; Dragnev, S.; Thurber, T.; Seed, B. A Comprehensive Collection of Experimentally Validated Primers for Polymerase Chain Reaction Quantitation of Murine Transcript Abundance. *BMC Genomics* **2008**, *9*, 633.
